# TrichoGate: An Improved Vector System for a Large Scale of Functional Analysis of *Trichoderma* Genes

**DOI:** 10.3389/fmicb.2019.02794

**Published:** 2019-12-10

**Authors:** Guillermo Nogueira-López, Fabiola Padilla-Arizmendi, Sarah Inwood, Sarah Lyne, Johanna M. Steyaert, Maria Fernanda Nieto-Jacobo, Alison Stewart, Artemio Mendoza-Mendoza

**Affiliations:** ^1^Bio-Protection Research Centre, Lincoln University, Lincoln, New Zealand; ^2^Department of Biochemistry, University of Otago, Dunedin, New Zealand; ^3^Lincoln Agritech Ltd, Lincoln, New Zealand; ^4^Plant & Food Research Gerald St, Lincoln, New Zealand; ^5^Foundation For Arable Research, Templeton, New Zealand

**Keywords:** Golden Gate cloning technology, *Trichoderma*, *Agrobacterium*-mediated transformation, carboxin resistance, TrichoGate, functional genomics, synthetic biology

## Abstract

Species of the genus *Trichoderma* are ubiquitous in the environment and are widely used in agriculture, as biopesticides, and in the industry for the production of plant cell wall-degrading enzymes. *Trichoderma* represents an important genus of endophytes, and several *Trichoderma* species have become excellent models for the study of fungal biology and plant–microbe interactions; moreover, are exceptional biotechnological factories for the production of bioactive molecules useful in agriculture and medicine. Next-generation sequencing technology coupled with systematic construction of recombinant DNA molecules provides powerful tools that contribute to the functional analysis of *Trichoderma* genetics, thus allowing for a better understanding of the underlying factors determining its biology. Here, we present the creation of diverse vectors containing (i) promoter-specific vectors for *Trichoderma*, (ii) gene deletions (using hygromycin phosphotransferase as selection marker), (iii) protein localization (mCherry and eGFP, which were codon-optimized for *Trichoderma*), (iv) gene complementation (neomycin phosphotransferase) and (v) overexpression of encoding gene proteins fused to fluorescent markers, by using the Golden Gate cloning technology. Furthermore, we present the design and implementation of a binary vector for *Agrobacterium*-mediated transformation in *Trichoderma* to increase the homologous recombination rate and the generation of a novel selection marker based on carboxin resistance.

## Introduction

Eukaryotic models, including ascomycetes fungi; for example, *Saccharomyces cerevisiae, Aspergillus nidulans*, and *Neurospora crassa*, have provided a comprehensive understanding of several biological processes that have been important for scientific research and applications (Nayak et al., 2006; Karathia et al., 2011; Zámborszky et al., 2014). Within the ascomycetes fungi, the genus *Trichoderma* has been widely used in agriculture and industry for its ability to display outstanding properties as biocontrol and biofertilizer agents (Druzhinina et al., 2011; Schmoll et al., 2016). *Trichoderma* spp. are distributed worldwide and more frequently found in the soil and/or rhizosphere, acting as free-living organisms. They can also colonize plant roots (Brotman et al., 2013), thus creating an endophyte-plant beneficial interaction. In general, the colonization of plant roots by *Trichoderma* spp. is beneficial to the host plant by enhancing plant growth and conferring resistance to biotic and abiotic stresses (Hermosa et al., 2012). In addition to the plant growth promotion abilities of *Trichoderma*, it also acts as a biocontrol agent of several plant pathogens (Rubio et al., 2014; Guigon-Lopez et al., 2015; Malmierca et al., 2016), diminishing signs and symptoms of the disease.

The availability of 25 genome sequences of *Trichoderma* spp.^1^ and^2^ has greatly assisted the genetic study of the *Trichoderma* genus (Mukherjee et al., 2013; Schmoll et al., 2016; Kubicek et al., 2019). However, the main challenge in genomics is to assign a function to predicted genes to reveal new insights into fungal biology. Functional gene characterization involves, in addition to in the generation of gene knockouts, studies of protein localization, identification of interaction partners, gene overexpression and complementation studies of the gene in question. Gene disruption by the substitution of gene sequences via homologous recombination is one of the most popular strategies to start the characterization of genes (Kück and Hoff, 2010).

To study gene function, the scientific community generally relies on the construction of recombinant DNA molecules using conventional cloning methodology that is based on restriction-digestion and ligation procedures. Although this strategy has been utilized to explore gene function with the generation of vectors to create deletion and/or over-expression mutants of the target genes, this technique has several disadvantages (e.g., time-consuming and retention of restriction endonuclease sites) when multi-targeted DNA fragments are ligated and inserted step-by-step into the vector.

The efficiency of homologous recombination during transformation in filamentous fungi is very low; usually less than 5% (Kück and Hoff, 2010) and the existing resistance markers are limited, and not all screening markers are useful for filamentous fungi. To overcome these limitations in *Trichoderma*, a streamlined knockout-construction system was created that included the construction of vectors using yeast mediated electroporation and the transformation of a *T. reesei* strain deficient in non-homologous end joining (Δ*tku*70), using three different selection markers (the hygromycin phosphotransferase *hph* gene, acetamidase-encoding *amdS* gene, and the *pyr4* gene encoding orotidine-5′-monophosphate decarboxylase) (Schuster et al., 2012).

The increase of functional genomics studies in the last decade has led to the development of more efficient and accurate cloning techniques that overcome the main issues of conventional cloning procedures including the Gateway and the Golden Gate cloning systems (Hartley et al., 2000; Walhout et al., 2000; Engler et al., 2008). The Gateway cloning system has been utilized for the analysis of functional genes *in planta* (Curtis and Grossniklaus, 2003) and the identification of functional genes during plant–microbe interactions. For example, in the fungus *Magnaporthe oryzae*, the creation of pFPL vectors using the Gateway system has facilitated the localization of effector proteins during plant infection (Gong et al., 2015). Nevertheless, one of the limitations of the Gateway system compared to the Golden Gate strategy is the retention of specific recombination site sequences in the final construct (Reece-Hoyes and Walhout, 2018). The Golden Gate cloning theory reported by Engler et al. (2008) allows for the creation of vectors containing more than two molecules in a single restriction-ligation reaction with the use of type IIs restriction enzymes; for example, *Bsa*I which cleaves outside of its recognition sequence 5′-GGTCTC-3′, resulting in 5′ or 3′ DNA overhangs that are four bases in length. This one-pot, one-step method creates recombinant vectors in less than one day compared with conventional cloning procedures which take several days (Engler et al., 2008) and permits the assembly of multiple DNA fragments with high efficiency (Engler et al., 2009; Weber et al., 2011). The Golden Gate cloning approach has been successfully implemented in plants (Emami et al., 2013; Lampropoulos et al., 2013), fungi (Terfrüchte et al., 2014; Sarkari et al., 2017; Hernanz-Koers et al., 2018; Egermeier et al., 2019) and bacteria (Wang et al., 2018).

Recently it was reported that codon usage strongly correlates with both protein and mRNA levels genome-wide in *Neurospora crassa*. Moreover, gene codon optimization in this fungus, resulted in strong up-regulation of protein and RNA levels, suggesting that codon usage is an essential determinant of gene expression (Zhou et al., 2016).

For the transformation of *Trichoderma* spp., several markers have been developed including auxotrophic markers (e.g., L-arginine, L-lysine and uridine biosynthesis pathways) that require an auxotrophic mutant as a parental strain (Baek and Kenerley, 1998; Jørgensen et al., 2014; Derntl et al., 2015). However, drug resistance markers have an advantage compared to auxotrophic markers by removing this restriction to a specific parental strain. In *Trichoderma*, selectable drug resistance markers have been developed for its transformation such as *hph* (hygromycin phosphotransferase) (Mach et al., 1994), *nptII* (neomycin phosphotransferase II, geneticin G418 resistance) (Gruber et al., 2012) and β*-tubulin-2* (benomyl resistance) (Peterbauer et al., 1992); however, resistance to these antimicrobial agents can differ between strains and species.

Succinate dehydrogenase (SDH) catalyses electron transfer from succinate to quinone during aerobic respiration (Singer et al., 1971). Carboxin is a specific inhibitor of this enzyme from several different organisms, including fungi (Broomfield and Hargreaves, 1992; Keon et al., 1994; Topp et al., 2002; Kilaru et al., 2009; Shima et al., 2009, 2011). Carboxin binds to the enzyme SDH, preventing electron transfer, and effectively killing the organism, as it cannot generate energy. Direct mutagenesis of the SDH gene, confers resistance to carboxin as described in other fungal models, including *Ustilago maydis* (Broomfield and Hargreaves, 1992; Keon et al., 1994; Topp et al., 2002), *Aspergillus oryzae* (Shima et al., 2009; Shima et al., 2011), and *Coprinopsis cinerea* (Kilaru et al., 2009), making it suitable as a drug resistance marker in fungi.

In this study, we aimed to improve a vector system for cloning and expression of any desired gene to study *Trichoderma’s* biology by using the Golden Gate strategy. The creation of entry vectors that contain specific molecules such as promoters, markers, terminators, tags and resistance cassettes were used for the generation of vectors to achieve gene deletion, protein localization and gene complementation. This was coupled with *Agrobacterium*-mediated transformation (AMT) to increase homologous recombination using *Trichoderma virens* as a model. Additionally, a new selection marker (carboxin resistance) was developed for the genetic transformation of *T. virens*.

## Materials and Methods

### Microorganisms, Media and Culture Conditions

*T. virens* Gv 29.8 (Kindly provided by Charles Kenerley, Texas A&M University, United States), *T. sp. “atroviride B”* LU132 (Braithwaite et al., 2017), and mutants were grown on potato dextrose agar (PDA) (Difco, Fisher Scientific, United States) at 25°C under a cycle of 12 h light and 12 h dark for 7 days to induce conidiation. Conidia were collected using sterile nanopure water and filtered through a double layer of sterile Miracloth (Millipore Merck, United States). The conidial concentration was determined using a Neubauer chamber. *Escherichia coli* cells transformants were grown on Luria Bertani (LB) medium supplemented with either 100 μg/mL ampicillin (PanReac AppliChem, Germany) or 50 μg/mL kanamycin (Sigma Aldrich, United States) at 37°C depending on the vector. *Agrobacterium tumefaciens* EHA105 cells transformants were grown on LB medium containing 25 μg/mL kanamycin and 25 μg/mL rifampicin (Sigma Aldrich, United States) at 28°C.

### Improvements in Codon Usage in *Trichoderma*

Codon usage and GC content of *T. virens* and *T. atroviride* were taken from the Codon Usage Database^3^ and provided by GenScript^®^ (Piscataway, NJ, United States) to codon-optimized the following the fluorescent markers eGFP, mCherry, and 3X eGFP, and epitope tags 3X c-Myc, 3X FLAG and 3X HA by using the OptimumGeneTM algorithm. These sequences were cloned into in the pUC57 cloning vector via *Eco*RV. Any restriction sites present in the multiple cloning site (MCS) from the pBluescript II KS (±) in the optimized sequences were removed (Supplementary Material).

### Creation of Entry Vectors

To create the TrichoGate entry vectors, synthetic genes produced by GenScript (eGFP, mCherry, 3X-eGFP, 3X-HA, 3X-FLAG, and 3X-c-Myc), promoters [translation elongation factor EF-1 α (*tef1*α) promoter and glycerol-3-phosphate dehydrogenase (*gpdh*) promoter], terminators [nopaline synthase terminator (*T-nos*) and tryptophan synthase transcription terminator (*TtrpC*)] and resistant cassettes were amplified by PCR using specific primers (Supplementary Table 1). These specific primers contained *Bsa*I recognition sequences and specific four bp overhang flanking sequences (5′-GGTCTC-n-NNNN-3′) which were introduced in front of the specific forward primer sequences (Supplementary Table 1). The sequence, GGTCTC is the *Bsa*I recognition site, while NNNN are the specific overhangs (indicated in color in the Supplementary Tables). The nucleotide (n) represents the extra-base between *Bsa*I and the specific overhang. For the reverse primers, the same sequence (5′-GGTCTC-n-NNNN-3′) was added in front of the reverse complement of the sequence of interest (Supplementary Tables 1, 2).

PCR products were amplified using high-fidelity PrimeSTAR HS DNA Polymerase (Takara, Japan) with the following cycling conditions: 98°C for 1 min; followed by 35 cycles of 98°C for 10 s; 60°C for 15 s; 72°C for 2 min, and a final extension at 72°C for 10 min. PCR products were purified using Wizard SV Gel and PCR Clean-Up System (Promega, United States). The purified molecules were quantified using a NanoDrop (NanoDrop Technologies Inc., United States), and cloned into the pICH41021 vector, a derivative of pUC19 with a mutated *Bsa*I recognition sequence in the ampicillin resistance cassette (kindly provided by Sylvestre Marillonnet), or pCR-Blunt vector (Thermo Fisher Scientific, United States). The ligation reaction was carried out in a total volume of 20 μL containing: 1 μL (5U) of T4 DNA ligase (Thermo Fisher Scientific, United States), 2 μL of 10X T4 DNA ligase buffer, 100 ng of linear vector pICH41021 (previously linearized with *Sma*I) or pCR-Blunt vector as manufacturer’s instructions, and 200 ng of the purified molecule (1:2 ratio of vector:insert). The recombinant vectors were transformed into *E. coli* TOPO F’ using chemically competent cells. Selection of positive colonies was performed on LB plates supplemented with 100 μg/mL ampicillin, IPTG (Zymo Research, United States) and X-Gal (PanReac AppliChem, Germany). The vectors were isolated, quantified and digested with *Bsa*I to verify the insertion and vector fragment sizes before sequencing.

### Creation of Destination pTrichoGate-1 Vector

pTrichoGate-1 was created by the substitution of the hygromycin resistance cassette from pYT6, a derivative plasmid from pCAMBIA1380 (Tanaka et al., 2007), with the *lacZ* ß-galactosidase gene from pAGM1311 (Addgene, United States). First, we amplified two PCR fragments using pYT6 as a template and Phusion Taq polymerase (NEB, United Kingdom). The PCR products were flanked by *Acc*651 and *Bsa*I restriction sites (with different four overhangs and compatible with the Bsa*I* sites flanking *lacZ* in pAGM1311). Primers oAM-LU-700 and oAM-LU-701 bind near to the *Bsa*I site in pYT6 and contained two point mutations that eliminate the *Bsa*I recognition site (GGTCTC) and created a new *Acc*651 recognition site (GGTACC) (Supplementary Table 2). Primers oAM-LU-696 or oAM-LU-695 were, however, designed to include the *Bsa*I restriction site with compatible 4 nucleotide overhangs to directionally subclone the *lacZ-Bsa*I fragment obtained from pAGM1311 (Supplementary Table 2). Thus the primer combination oAM-LU-700 and oAM-LU-696 produced a 3,578 bp PCR product while the combination oAM-LU-701/oAM-LU-695 generated a 2,852 bp PCR product. The PCR were obtained by using the following cycling conditions: 98*°*C for 1 min; followed by 35 cycles of 98*°*C for 30 s; 60*°*C for 15 s; 72*°*C for 2 min, and a final extension at 72*°*C for 10 min. These PCR products were cut with *Bsa*I and *Acc*65I, then purified with the Wizard SV Gel and PCR Clean-Up System (Promega, United States) and ligated together with the 596 bp *lacZ-*Bsa*I* fragment obtained from pAGM1311 by T4 DNA ligase (Thermo Fisher Scientific, United States). The resulting 6,990 bp ligation vector (called pTrichoGate-1 vector) was transformed into *E. coli* and verified with restriction enzymes and sequencing.

### Creation of Final Vectors

Destination vectors pAGM1311 (Addgene, United States) or pTrichoGate-1 were used to insert the desired construct to create the final vectors. Both vectors contain the *lacZ* ß-galactosidase gene bordered by *Bsa*I restriction sites and the kanamycin resistance gene for selection in *E. coli* (Supplementary Figure 1). One-tube restriction-ligation reactions were set up independently for each construct as follows: in a total volume of 10 μL containing 10 U of *Bsa*I-HF (NEB, United Kingdom), 10 U T4 DNA ligase (Thermo Fisher Scientific, United States), 1 μL of 10X T4 DNA ligase buffer, (∼80 ng) PCR products [in the case of Sm1, TvTF1 (ORFs) and terpene synthases flanks] and entry vectors (∼75 ng) containing DNA fragments and destination vector (∼50 ng) were cut and ligated in the same reaction. The reaction was incubated for 2 min at 37°C and 5 min at 16°C; both steps were repeated 40 times, followed by final digestion for 5 min at 50°C and then heat inactivation for 5 min at 80°C. The recombinant vectors were transformed into *E. coli* chemically competent cells using 10 μL of restriction-ligation product. Selection of positive colonies was performed on LBA plates supplemented with 50 μg/mL kanamycin (Sigma Aldrich, United States), IPTG and X-gal. The vectors from positive *E. coli* colonies were isolated and verified by digestion with *Eco*RV or *Bsa*I enzymes. DNA fragments were separated by 0.8% agarose gel electrophoresis. The vectors were sequenced before being used for *Trichoderma* protoplast transformation (i.e., using linearized vectors).

For pTrichoGate-1 derivative vectors (e.g., knockout construct vectors), electrocompetent cells from *A. tumefaciens* EHA105 were used. Electroporation was carried out, using the “Agr” setting (cuvette 0.1 mm, 2.2 kV and single pulse) as per the manufacturer’s instructions on a MicroPulser Electroporator (Bio-Rad, United States). The resulting transformants were selected on LB medium containing 25 μg/mL kanamycin and 25 μg/mL rifampicin. Before the fungal transformation, the pTrichoGate-1 derivative plasmids cloned in *Agrobacterium* were re-isolated and re-confirmed as previously described.

#### Knockout Vectors

For the gene knockout strategy, putative terpene synthase (TS) encoding genes (called here TS1 and TS2) from *T. virens* were chosen as an example. PCR primers were designed to amplify 1–1.3-kb of the flanking regions of the gene of interest (Supplementary Table 1). The PCR reaction was carried out using Phusion Taq Polymerase (Thermo Fisher Scientific, United States) with the following cycling conditions: 98°C for 1 min; followed by 35 cycles of 98°C for 5 s, 62°C for 15 s and 72°C for 1 min; and a final extension at 72°C for 10 min. Each primer combination (Supplementary Table 3) was designed to produce PCR products having flanking *Bsa*I restriction sites to generate appropriate four base overhangs for the correct orientation for the ligation of the hygromycin B phosphotransferase gene (*hph*) from pTrichoGate-21 and the destination vector pTrichoGate-1 (Table 1 and Supplementary Figure 2). The PCR products from both borders corresponding to the desired gene to be knocked out were purified, quantified and then combined with pTrichoGate-1 and pTrichoGate-21; the one-tube restriction-ligation reaction performed as described above (Supplementary Figure 2).

**TABLE 1:** Entry and destination vectors optimized for functional genetics in *Trichoderma.*

**Vector name**	**Description**	**Bacterial resistance**	**NCBI Accession number**
**Empty destination vector**			
pTrichoGate-1	pYT6-lacZ-*Bsa*I	Kanamycin	MN007105
***Trichoderma* promoters**			
pTrichoGate-2	*gpdh* promoter	Ampicillin	MN007106
pTrichoGate-3	*tef1*α promoter	Ampicillin	MN007107
**Coding sequences**			
pTrichoGate-4	Synthetic mCherry	Ampicillin	MN007108
pTrichoGate-5	Synthetic eGFP	Ampicillin	MN007109
pTrichoGate-6	Synthetic 3X eGFP	Ampicillin	MN007110
**N-terminal tags**			
pTrichoGate-7	Synthetic mCherry-linker	Ampicillin	MN007111
pTrichoGate-8	Synthetic eGFP-linker	Ampicillin	MN007112
pTrichoGate-9	Synthetic 3X eGFP-linker	Ampicillin	MN007113
**C-terminal tags**			
pTrichoGate-10	Linker-synthetic mCherry	Ampicillin	MN007114
pTrichoGate-11	Linker-synthetic eGFP	Ampicillin	MN007115
pTrichoGate-12	Linker-synthetic 3X eGFP	Ampicillin	MN007116
pTrichoGate-13	Synthetic 3X Hemagglutinin tag (HA)	Ampicillin	MN007117
pTrichoGate-14	Synthetic 3X c-Myc tag peptide (EQKLISEEDL)	Ampicillin	MN007118
pTrichoGate-15	Synthetic 3X FLAG Peptide	Ampicillin	MN007119
**Terminators**			
pTrichoGate-16	T-nos terminator	Ampicillin	MN007120
pTrichoGate-17	TrpC terminator	Ampicillin	MN007121
**Fungal resistance cassettes**			
pTrichoGate-18	Hygromycin resistance cassette	Ampicillin	MN007122
pTrichoGate-19	Carboxin resistance cassette	Kanamycin	MN007123
pTrichoGate-20	Geneticin resistance cassette	Ampicillin	MN007124
pTrichoGate-21	Hygromycin resistance cassette for gene deletion	Ampicillin	MN007125

#### Fluorescent Marker Vectors

For the creation of the fluorescent marker vector, the vectors pTrichoGate-2 (*gpdh* promoter), pTrichoGate-4 (synthetic mCherry) and pTrichoGate-16 (nopaline synthase terminator, T-nos) and pTrichoGate-18 (hygromycin resistance cassette) were combined in a single tube with pAGM1311 and one-tube restriction-ligation reaction performed as described above, using 50 ng for each vector. The reaction was transformed into *E. coli* using chemically competent cells and the resulting plasmids isolated and analyzed by restriction enzymes before being used for *T. virens* protoplast transformation using the method described below.

#### Localization Vector

The open reading frame from the proteinaceous elicitor secreted protein Sm1 (ID 110852) that encodes a polypeptide of 138 amino acids with predicted mass of 14.4 kDa, an isoelectric point of 5.76 and contains a signal peptide of 18 amino acids (Djonovic et al., 2006); and the fungal transcription factor 1 (TvTF1) (ID 158742), which contains the classical nuclear localization signal, from *T. virens* were amplified by PCR using primer combination (Sm1 Loc_F and Sm1 Loc_R) and (TF Loc_F and TF Loc_R), respectively (Supplementary Table 1). The PCR reaction was carried out with Phusion Taq Polymerase (Thermo Fisher Scientific, United States) using *T. virens* DNA as template with the following cycling conditions: 98°C for 1 min; followed by 35 cycles of 98°C for 5 s, 62°C for 15 s and 72°C for 1 min; and a final extension at 72°C for 10 min. Reverse primers Sm1 Loc_R and TF Loc_R were designed to hybridize with the last 21 nucleotides of the Sm1 or TvTF1 gene without the last three nucleotides which form the stop codon. For the Sm1 localization, the following genetic material was combined in a single tube and the one-tube restriction-ligation reaction performed: the PCR product from *SM1*, the vectors pTrichoGate-2 (*gpdh* promoter), pTrichoGate-10 (Linker-synthetic mCherry), pTrichoGate-16 (T-nos terminator), pTrichoGate-18 (hygromycin resistance cassette) and pAGM1311. For the TvTF1 localization, the following molecules were used for the one-tube restriction-ligation reaction performed: the *tvtf1* PCR product, pTrichoGate-3 (*tef1*α promoter), pTrichoGate-5 (Linker-synthetic eGFP), pTrichoGate-16 (T-nos terminator), pTrichoGate-18 (hygromycin resistance cassette) and pAGM1311 (Supplementary Figure 2). The fluorescent markers (mCherry and eGFP) from the vectors pTrichoGate-10 and pTrichoGate-11 were designed to contain a glycine and serine-rich flexible linker (GSAGSAAGSGEF) located at the N-terminus of the synthetic mCherry or synthetic eGFP (Waldo et al., 1999; Chen et al., 2013). The one-tube restriction-ligation reactions were transformed into *E. coli* using chemically competent cells and the plasmid isolated and analyzed by restriction enzymes before *T. virens* protoplast transformation.

#### Complementation Vector

To complement the 2-oxoglutarate (2OG)-Fe dioxygenase 1 (Tv2OG1) in the *T. virens* knockout mutant genome (Nogueira-Lopez, et al., in preparation), primers (Comp2OG_F and Comp2OG_R) were designed using the published *T. virens* genome database to amplify approximately 1.5 kb native promoter and 0.5 kb terminator sequences from the target gene region with the addition of a *Bsa*I restriction site located on the 5′ and 3′ end flanks (Supplementary Table 1). Genomic DNA from *T. virens* was used as template. The complementing region was ligated to pAGM1311 and pTrichoGate-20, which contains the neomycin phosphotransferase encoding gene (*nptII*) (conferring geneticin G-418 resistance and termed GtR) under the control of the tryptophan gene promoter (*PtrpC*) and terminator (TtrpC) (Supplementary Figure 2). The reaction was transformed into *E. coli* using chemically competent cells and the plasmid isolated and analyzed by restriction enzymes before being used for *T. virens* protoplast transformation.

### Carboxin Resistance Cassette

Direct mutagenesis of *T. virens* succinate dehydrogenase-encoding *sdhTv* gene (Protein ID88418, JGI *T. virens*) to confer resistance to carboxin was performed as described by Broomfield and Hargreaves (1992) in *U. maydis*. For the creation and implementation of the carboxin resistance cassette with Golden Gate methodology, five-point mutations were generated to eliminate four internal *Bsa*I restriction sites in the carboxin resistance cassette sequence and to create the point mutation in the *sdhTv* iron-sulfur subunit protein (Ip) subunit (SdhB) that substitutes the His (CAC) codon for Leu (CTC) in codon 246, conferring resistance to carboxin (Broomfield and Hargreaves, 1992) (Supplementary Figure 3). Double-joint PCR methodology described by Yu et al. (2004) with modifications was used to eliminate *Bsa*I sites and generate codon substitution by adding nucleotide changes in the primers. First-round PCR: amplification of 5′- and 3′ regions; six independent PCR products with 23–31 bases of homologous sequence overlapping with the ends of the next PCR product (for the primer combination, see Supplementary Figure 3A). Second-round PCR: fusion of six fragments into three using flanking primer combinations (CbxR1-CbxR4, CbxR5-CbxR8) for each independent PCR product. Third round PCR: amplification of the fused fragments into one by using LF and RF flank primers (CbxR1-CbxR12) (Supplementary Figure 3). PCR products were amplified using Phusion High-Fidelity DNA Polymerase (Thermo Fisher Scientific, United States) with the following cycling conditions: 98°C for 1 min: followed by 35 cycles of 98°C for 10 s; 60°C for 30 s; 72°C for 30 s, and a final extension at 72°C for 10 min. The carboxin resistance cassette was subcloned into pCR-Blunt vector, linearized with blunt ends (Thermo Fisher Scientific, United States) according to the manufacturer’s instructions.

### Nucleotide Sequence Accession Numbers

The DNA sequences of pTrichoGate-1 to pTrichogate-21 have been submitted to the GenBank database under accession numbers MN007105–MN007125 and are displayed in Table 1. These plasmids are available in Addgene^4^.

### Sensitivity of *T. virens* Conidia to Carboxin

The antibiotic sensitivity of *T. virens* wild-type (WT) to carboxin (Sigma Aldrich, United States) was evaluated using four different media: PDA medium, AY medium (144 mM acetate–acetic acid buffer at pH 6.5, 5 g l^–1^ yeast extract and 15 g l^–1^ bacteriological agar), EY medium (10 ml l^–1^ ethanol, 5 g l^–1^ yeast extract and 15 g l^–1^ bacteriological agar) and GY medium (10 g l^–1^ glucose, 5 g l^–1^ yeast extract and 15 g l^–1^ bacteriological agar) (Shima et al., 2009). Carboxin resistance was tested on the different media mentioned above supplemented with different concentrations of carboxin (0, 50, 150, and 250 μg carboxin, diluted in DMSO per mL of medium). *T. virens* WT conidia (1 × 10^6^) were homogeneously spread onto plates contacting the media mentioned above and incubated at 25°C for 7 days under a light/dark cycle. The samples were observed using a stereomicroscope (SZX12 Olympus, Japan) to confirm growth and germination inhibition. For each analysis, three replicates were used with different antibiotic concentrations.

### Protoplast Transformation

The strategy used for *Trichoderma* protoplast transformation is described in the Supplementary Material. For the transformation of *Trichoderma* protoplasts, 10 μg of DNA containing the desired linearized vector was used. The digested vectors were visualized to confirm single digestion and then purified using Wizard SV Gel and PCR Clean-Up System (Promega, United States). For *T. virens*, positive transformants were recovered on recovering medium PDA (PDB supplemented with 0.7% agar and 0.7M Sucrose) amended with 100 μg/mL hygromycin, Vogel’s medium (supplemented with 0.7% agar) with 750 μg/mL geneticin G418 or solid AY-medium (supplemented with 0.7% agar and 0.7 M Sorbitol) with 350 μg/mL carboxin. For *T. atroviride*, the transformants were recovered on PDA amended with 100 μg/mL hygromycin. *Trichoderma* mutants were single spore purified as follows: conidia from *Trichoderma* positive mutants were grown on PDA supplemented with antibiotics for 5 days at 25°C under a 12 h day/night cycle to induce conidiation. To obtain mononucleate mutants, aliquots of conidial suspensions (1 × 10^3^ cells, 100 μL) were plated on PDA amended with antibiotics and plates were incubated in darkness. After 18 h, colonies were observed using a stereomicroscope. A single colony was selected and inoculated to PDA with antibiotics. Single spore purification was repeated ten times to ensure the isolation of mononucleate strains. To confirm the stability of the mutation in *Trichoderma* mutants, all confirmed mutants were sub-cultured (10 times) on PDA without antibiotics and then transferred to PDA with antibiotics. The DNA of stable mutants was isolated using standard methodologies, and then confirmed by PCR and/or Southern blot. This methodology was used for the genetic transformation of *T. virens* with a fluorescent marker, localization, complementation and novel resistance marker vectors, but also for *T. atroviride* LU132 with mCherry.

### *Agrobacterium*-Mediated Transformation

*Agrobacterium*-mediated transformation with modifications was performed for the generation of the *T. virens* knockout mutants and according to the method reported by de Groot et al. (1998), with improvements as described in Zeilinger (2004). After vector verification, 0.1 mL of pre-cultured *A. tumefaciens* cells containing desired vectors were inoculated into the minimal medium (MM) [10 mM K_2_HPO_4_, 10 mM KH_2_PO_4_, 2 mM MgSO4, 2.5 mM NaCl, 0.7 mM CaCl_2_, 10 mM D-glucose, 9 μM FeSO_4_, 4 mM(NH_4_)_2_SO_4_] supplemented with 25 μg/mL kanamycin and 25 μg/mL rifampicin and incubated overnight at 28°C with shaking at 250 rpm. The culture was centrifuged at 3,000 × *g* for 10 min at room temperature. *A. tumefaciens* cells were re-suspended in 50 mL of induction media with acetosyringone (IMAS broth) [MM, MES buffer (40 mM), 50% glycerol, acetosyringone] to an optical density of OD_660_ = 0.15, and the culture was incubated for up to 4 h at 28°C with shaking at 200 rpm until OD_660_ of 0.3 was reached. *Trichoderma* protoplasts were created according to the method described in the Supplementary Material. Equal volumes containing 100 μL of each *A. tumefaciens* culture and *T. virens* protoplast suspension (1 × 10^6^ protoplasts) were plated onto sterile cellophane placed on IMAS agar and incubated at 23°C for 48 h in complete darkness. The circular cellophane was cut into eight triangular size pieces and transferred to PDA plates supplemented with 100 μg/mL hygromycin B and 300 μg/mL Timentin (GlaxoSmithKline, United Kingdom), and then incubated at 25°C for 2 days in complete darkness. *T. virens* colonies grown after incubation were transferred to PDA amended with antibiotics and subsequently purified to mitotic stability by ten rounds of single-spore isolation.

### Culture Conditions for Protein Extraction

*T. virens* (WT and Sm1-Linker-mCherry) conidia, were inoculated onto PDA plates and incubated at 25°C with a 12 h day/night cycles for 7 days. Conidia were harvested as described above. The conidial suspension (1 × 10^6^ conidia) was inoculated into 100 mL flasks containing 50 mL of PDB and incubated for 72 h at 25°C with shaking (160 rpm). The mycelia from the culture media were harvested by filtration and washed three times using sterile nanopure water, and then frozen in liquid nitrogen and stored at −80°C until protein extraction. The supernatant (50 mL) from the culture media was filtered through a double layer of sterile Miracloth and then filtered again through a 0.45 μm nylon filter (ReliaPrep, Ahlstrom-Mujnksjö, Finland) to remove any mycelia. The collected supernatant was stored at −80°C until protein extraction.

### Protein Extraction

#### Mycelia Protein Extraction

For total protein extraction from *T. virens* mycelia, the methodology described by Bhadauria and Peng (2010) was implemented with modifications. Frozen mycelia (0.5 g) were ground into a fine powder using liquid nitrogen in precooled conditions. The powder was homogenized in 5 mL of ice-cold Tris/EDTA extraction buffer, containing 10 mM Tris-HCL pH 8, 1 mM EDTA, 2% w/v polyvinylpolypyrrolidone (PVPP) and with 0.3% (v/v) Pefabloc (Sigma Aldrich, United States), and then samples were centrifuged at 5,000 × *g* for 30 min at 4°C. The supernatant was collected, and proteins precipitated with 4 volumes of cold (−20°C) 10% w/v trichloroacetic acid (TCA)/acetone with 0.007% w/v DTT. Samples were centrifuged at 3,000 × *g* for 10 min; the pellet was washed three times with ice-cold acetone containing 0.007% w/v DTT. Protein pellets were dried to evaporate any remaining acetone and resuspended in rehydration buffer containing 7 M Urea, 2 M thiourea, 20 mM DTT and 2% w/v Triton X-100.

#### Supernatant Protein Extraction

For protein extraction of the *T. virens* secreted fraction, the methodology described by Medina and Francisco (2008) was implemented with modifications. For protein precipitation, 20 mL of broth supernatant was mixed with an equal volume of cold 20% (w/v) TCA solution. The mixture was incubated for 2 h at −20°C to allow protein precipitation and then centrifuged for 10 min at 14,000 × *g*. The supernatant was decanted, and the pellet was washed with 1 mL of cold 70% ethanol. The samples were vortexed and centrifuged for 3 min; this step was repeated three times. The final pellet was dried with the addition of 1 mL of acetone; the samples were vortexed and centrifuged for 1 min. The acetone was decanted, and the pellet air-dried for 30 min and then resuspended in the rehydration buffer as described above.

#### Western Blot

For Western blot analysis, 20 μg of protein was loaded into each well of a precast SDS-PAGE gel (Bio-Rad, United States), along with a molecular weight marker (PageRuler Plus Prestained Protein Ladder, Thermo Fisher Scientific, United States), and run at 100 V for 1 hr. Transfer of proteins to a PVDF blotting membrane (Amersham Hybond P; GE Healthcare Life Sciences, United Kingdom) was achieved as follows: (1) the gel was placed in 1 X transfer buffer pH 8.3 (25 mM Tris, 190 mM glycine and 20% methanol); (2) the transfer and the development was performed as described in a general protocol for western blotting (Bio-Rad, United States). For antibody incubation, the membrane was blocked in 5% skim milk in Tris-buffered saline Tween 20 buffer (TBST) (20 mM Tris pH 7.5, 150 mM NaCl and 0.1% Tween 20). The membrane was then incubated with the addition of 1:10,000 (v/v) anti-mCherry or anti-eGFP antibodies (BioVision, United States) dilution in TBST. The blot was rinsed in TBST and then incubated with 1:10,000 (v/v) HRP-linked secondary antibody (Cell Signaling Technology, United States) solution, using TBST for the dilution. A chemiluminescent substrate (500 μL luminol enhancer + 500 μL peroxide solution) was applied to the blot according to the manufacturer’s recommendations (Amersham ECL Prime Western Blotting Detection Reagent; GE Healthcare Life Sciences, United Kingdom).

### Microscopic Visualization of *Trichoderma virens* Transformants

*T. virens* mutants (Sm1-Loc and TvTF1-Loc) were examined using fluorescent microscopy (Olympus BX51, Olympus, Japan). Colonies (3 days old) were incubated as described above. Fresh mycelia were analyzed under fluorescent microscopy to localize fluorescent protein mCherry and eGFP fusions within cells. Fluorescent proteins detection was observed by exciting at 488 and 561 nm. The detection wavelengths were in the range of 500–535 and 580–630 nm for eGFP and mCherry, respectively. Samples were analyzed using cellSens software (Olympus, Japan).

## Results

### TrichoGate Cloning Strategy

**FIGURE 1:**
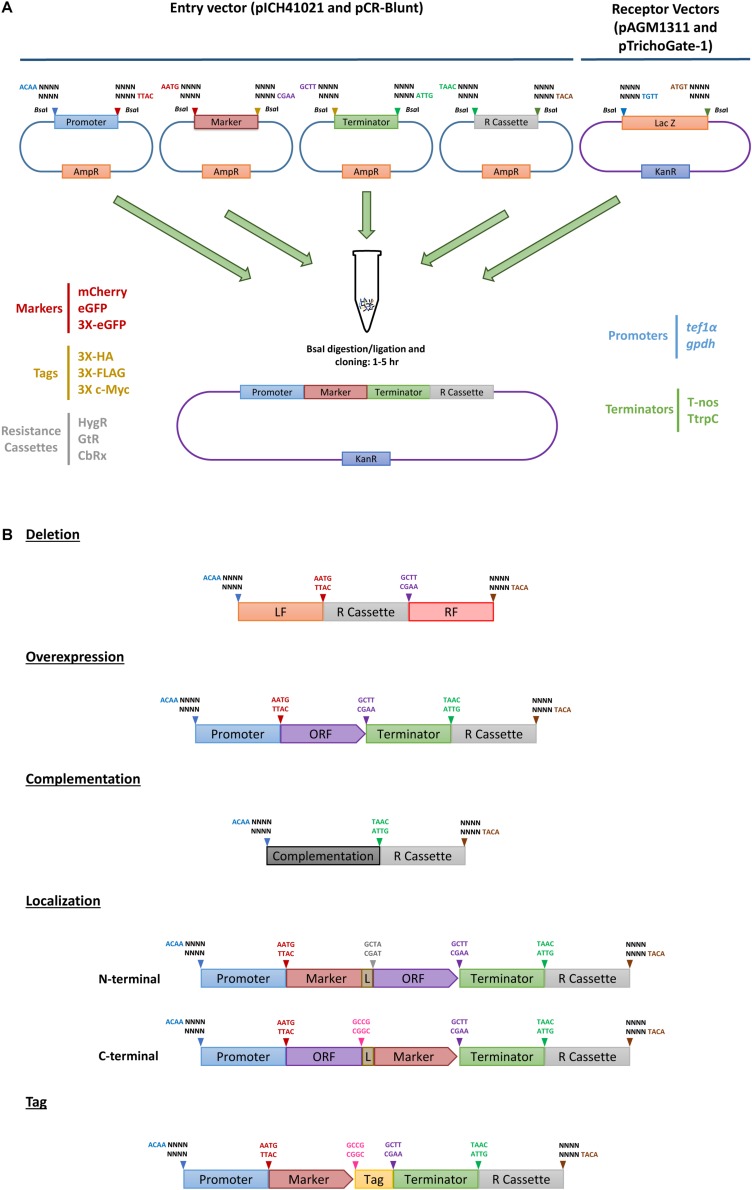
TrichoGate cloning system. Schematic representation of the TrichoGate cloning strategy to accomplish genomic modification in *T. virens*. **(A)** Implementation of TrichoGate cloning system using type II restriction enzyme (*Bsa*I), target DNA sequences containing *Bsa*I sites in their flanking regions with unique four bases overhangs must be subcloned in linearized entry vectors (pICH41021 or pCR-Blunt) carrying the ampicillin (AmpR) or kanamycin resistance (KanR) gene for selection in *E. coli*, respectively. TrichoGate vectors were constructed using up to five entry vectors (pICH41021 or pCR-Blunt) and/or PCR products containing synthetic DNA molecules of interest (e.g., promoters, markers, ORFs, terminators, tags and resistance cassettes) and one of the receptor vectors (pAGM1311 or pTrichoGate-1) that carry the KanR gene for selection in *E. coli*. *Bsa*I restriction-ligation was carried out in one single microcentrifuge tube containing target DNA molecules for up to 5 h. **(B)** Examples of hypothetically generated vectors using *Bsa*I restriction/ligation reaction for functional gene analysis in *T. virens* (e.g., gene deletion, gene complementation, overexpression, localization and immunoprecipitation).

**FIGURE 2:**
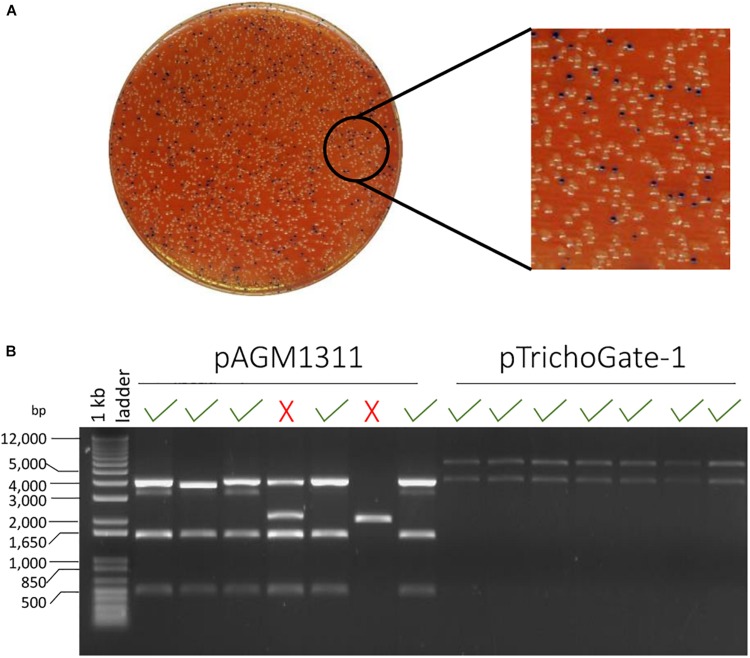
The efficiency of TrichoGate cloning system. **(A)** Luria-Bertani (LB) plate supplemented with 50 μg/mL kanamycin for selection of *E. coli* transformants containing constructs that were created using TrichoGate cloning system. Blue/white screening, *E. coli* cells transformed with vectors containing correct recombinant DNA should be white. **(B)** Restriction of plasmid DNA of minipreps from seven white colonies digested by *Eco*RV and run on a 1% agarose gel to verify insertion of the correct (√) or incorrect (X) construct by using two different receptor vectors: pAGM1311 and pTrichoGate-1. DNA from 70% (pAGM1311) and 100% (pTrichoGate-1) from white colonies analyzed have the desire restriction pattern.

The TrichoGate system was developed to create a simple, efficient and reliable toolbox that enables assembly of synthetic DNA molecules into cloning vectors for the transformation of *Trichoderma* by using the Golden Gate cloning strategy. In this study, entry vectors (pICH41021 or pCR-Blunt) were used as recipients of synthetic DNA molecules (promoters, markers, tags, terminators and resistance cassettes) that were designed to be flanked by type II recognition sites by PCR. Each synthetic DNA molecule was flanked by a specific four nucleotide overhang region at the 5′- and 3′-end that was complementary to the next overhang in the next molecule to define the orientation and position in the final construct (Figure 1). To ensure a sufficient expression level of markers (mCherry, eGFP, 3X eGFP) and tags (3X HA, 3X FLAG and 3X c-Myc), we codon-optimized using the Codon Usage Database^5^ (see Tables in Supplementary Material). Chemically competent *E. coli* cells were transformed with the entry vectors containing target DNA molecules. All molecules used in this study, including promoters (*tef1*α and *gpdh*), markers (mCherry, eGFP, 3X eGFP, N-linker-mCherry, mCherry-linker-C, N-linker-eGFP, eGFP-linker-C, N-linker-3X eGFP and 3X eGFP-linker-C), tags (3X HA, 3X FLAG and 3X c-Myc), terminators (T-nos and TtrpC) and resistance cassettes (HygR, GtR and CbxR) were successfully subcloned into entry vectors (pICH41021 or pCR-Blunt) and ≥80% of the clones analyzed showed the correct construct (data not shown). To corroborate the efficiency and viability of the TrichoGate system in *Trichoderma*, these modules were used for the generation of different cloning approaches to study functionality of the selected proteins in *Trichoderma*: (i) gene deletion, (ii) gene overexpression, (iii) gene complementation, (iv) protein localization using fluorescent markers and (v) protein tagging with non-fluorescent markers. Each restriction-ligation was performed with their independent modules previously described above. Transformation of *E. coli* cells with the restriction-ligation products of the six independent constructs showed that (≥60%) of positive *E. coli* colonies contained the desired construct by using either pAGM1311 or pTrichoGate-1 as a receptor vector (Figure 2 and Supplementary Figure 2).

### Deletion and Complementation Studies in *T. virens*

#### Deletion of *T. virens* Terpene Synthases

**FIGURE 3:**
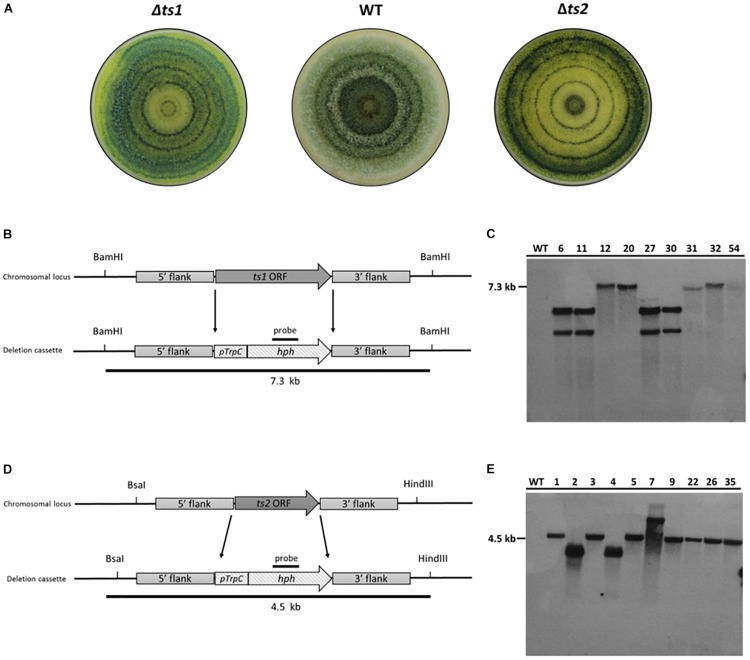
Confirmation of homologous recombination event of the HygR cassette in deletion mutants by Southern blot. **(A)** Colony appearance growing on axenic media (PDA) under light/dark cycle. **(B–D)** Schematic representation of gene deletion strategy. The ORF of each targeted gene is replaced by the homologous integration of the PtrpC/*hph* fragment (1.4 kb) that confers resistance to hygromycin B. **(B)** A 7.3 kb fragment would result for the *ts1* deletion mutants when digested with *Bam*HI. **(C)** Southern blot analysis of *T. virens* WT strain and *ts1* deletion mutants (6, 11, 12, 20, 27, 30, 31, 32, and 54) **(D)** A 4.5 kb fragment would result for the *ts2* deletion mutants when digested with *Bsa*I and *Hin*dIII. **(E)** Southern blot of *T. virens* WT strain and *ts2* deletion mutants (1, 2, 3, 4, 5, 7, 9, 22, 26, and 35). Expected fragments sizes for each deletion event along the left side of the films.

The deletion of two terpene synthases (*ts1* and *ts2*) encoding genes of *T. virens* was performed by homologous recombination replacing the open reading frame of each targeted gene with the PtrpC/*hph* fragment using *Agrobacterium*-mediated transformation. A total of 25 stable transformants of each deletion, were screened for their capability of continuously growing in PDA plates supplemented with 100 μg/mL hygromycin B and stable transformants from each deletion were screened for the insertion of the *hph* by PCR (Supplementary Figure 4 and data not shown). Nine and ten stable transformants of potential TS1 and TS2 deletion mutants, respectively, were randomly selected to confirm homologous recombination of the HygR cassette by Southern Blot. The hybridization results of the labeled *hph* probe showed a single band (7.3 kb) in five TS1 mutants when gDNA was digested with *Bam*HI, and a single band (4.5 kb) was shown in seven TS2 mutants when gDNA was digested with *Bsa*I and *Hind*III; thus, validating the replacement of the native genes with the HygR cassette. No fragments were shown for their parental strain (Figure 3). Overall, these results demonstrate that by using the Golden Gate cloning strategy coupled with *Agrobacterium*-mediate transformation, we were able to obtain a percentage of homologous recombination above 50%.

To prove homologous integration on both the 5′ and 3′ ends of the deletion constructs, a couple of additional PCR reactions was make. For the verification of the double recombination in the 5′ region, the primer combination TS1 Ext-LF/oAM-LU217 or TS2 Ext-LF/oAM-LU217 (Supplementary Table 1 and Supplementary Material) generated a PCR product in the positive recombinants. For the verification of the 3′ double recombination the primer combination oAM-LU218/TS1 Ext RF or combination oAM-LU218/TS2 Ext RF (Supplementary Table 1 and Supplementary Material) were used instead (Supplementary Figure 4).

### Complementation of *T. virens* 2OG-Fe Dioxygenase

**FIGURE 4:**
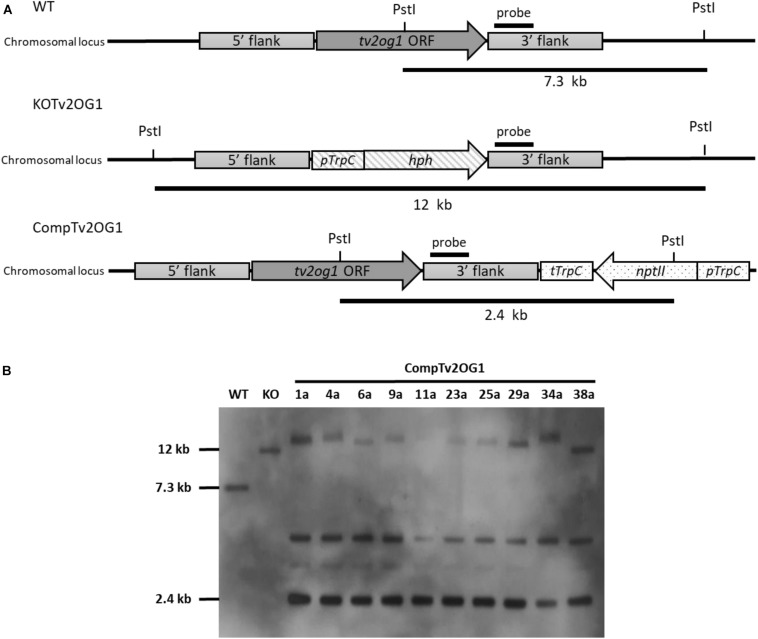
Southern blot confirmation of complementing mutants of the tv2og1 gene in *T. virens*. **(A)** Schematic representation of the gene deletion and complementation strategy. For gene deletion, *tv2og1* was replaced by the homologous integration of the *pTrpC/hph* fragment (1.4 kb) that confers resistance to hygromycin B. For gene complementation, the Δ*tv2og1* null mutant was transformed with the complementing construct created using the TrichoGate system. The complementing region containing a 1.5 kb promoter and 0.5 kb terminator fragments, along with the *tv2og1* ORF, was fused to the neomycin phosphotransferase encoding gene (*nptII*) (conferring resistance to geneticin G-418) under control of the tryptophan gene promoter (*pTrpC*) and tryptophan gene terminator (*tTrpC*). **(B)** Southern blot of the *T. virens* WT and the *tv2og1* deletion and complementing mutants was carried out using a fragment of *tv2og1* terminator as a probe. The blot shows the 2.4 kb fragment size expected in *T. virens* WT strain, the 12 kb fragment size expected for the *T. virens tv2og1* null mutant strain and the 2.5 kb fragment expected in *T. virens Ctv2og1* strains when gDNA was digested with *Pst*I. An extra band was present in all ten complementing mutants suggesting ectopic integrations of the GtR cassette in the *T. virens* genome.

The complementation of the 2OG-Fe dioxygenase (*tv2og1*) encoding gene of *T. virens* was achieved by inserting the WT allele into the *T. virens* Δ*tv2og1* strain (Nogueira-Lopez et al., in preparation). The complementing construct contained the neomycin phosphotransferase encoding gene (*nptII*) that conferred resistance to geneticin G-418 and was used as a selectable marker. Twenty-five transformants were screened for the ability to grow continuously and sporulate in Vogel’s medium supplemented with 750 μg/mL geneticin G-418. Genetic analysis (Southern blot) of ten randomly selected transformants was performed to corroborate the insertion of the *tv2og1* ORF and GtR cassette (Figure 4). DNA from the WT and Δ*tv2og1* strains was used as a positive and negative control, respectively. The hybridization results of DNA previously digested with *Pst*I showed that all screened transformants were identified as positive recombinants, confirming the presence of the *tv2og1* gene and GtR (2.4 kb band). However, all contained more than one ectopic integration of the *tv2og1* complementing construct (Figure 4). The WT and Δ*tv2og1* showed the expected fragment sizes of 7.3 and 12 kb, respectively. These results confirmed the integration of the complementation construct into the *T. virens* genome by combining TrichoGate vectors and protoplast transformation.

### Expression of Codon-Optimized mCherry in *Trichoderma*

**FIGURE 5:**
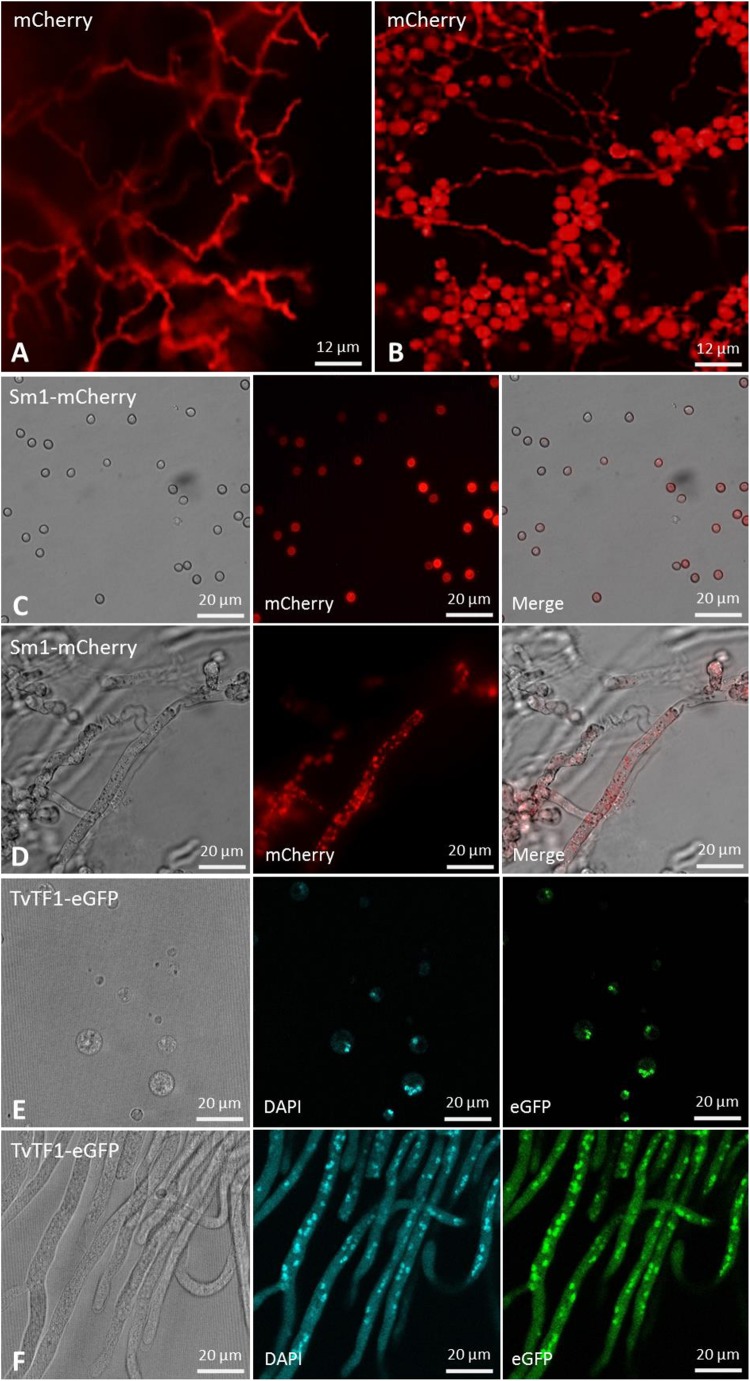
Microscopic observation of the *T. virens* mutants transformed with a fluorescent marker or protein localization vector generated through the TrichoGate system. Fluorescent microscopy of *T. virens* mycelia and conidia to verify the expression of the mCherry fluorescent marker under the control of the glyceraldehyde 3-phosphate dehydrogenase (*gpdh*) promoter from *T. virens*. Red fluorescence corresponding to the mCherry marker observed in *T. virens* **(A)** mycelia and **(B)** conidia. Microscopic observation of the Sm1-linker-mCherry expressed in *T. virens* **(C)** conidia and **(D)** mycelia; left bright-field, middle fluorescence and right merged images. Microscopic observation of the TvTF1-linker-eGFP expressed in *T. virens* **(E)** protoplasts and **(F)** mycelia; left bright-field and right fluorescence images. To visualize mCherry, 587 nm of excitation and 610 nm emission were used; while for the eGFP 484 nm excitation and 507 nm emission were used. No autofluorescence was observed in *T. virens* mycelia and conidia using either red or green channels. were visualized using an Olympus BX51 compound microscope. Images were captured using an Olympus DP70 digital camera system and processed with the CellSens software (Olympus).

The validation of the TrichoGate technology was first analyzed by testing genetic transformation of *T. virens* protoplasts with a linearized TrichoGate vector containing the *gpdh* promoter, codon-optimized mCherry, T-nos terminator and HygR cassette. Stable *T. virens* hygromycin resistant colonies that were able to grow in the presence of 100 μg/mL hygromycin were screened, and the presence of the *hph* gene was verified by PCR (data not shown). Five-day old cultures of *T. virens-*mCherry strains were used to observe mCherry expression in both conidia and mycelia under a fluorescence microscope. The expression of mCherry as the reporter gene under the control of the constitutive *gpdh* promoter was visualized in *T. virens* (Figures 5A,B). Remarkably, the mCherry-tagged colonies displayed intense cytosolic fluorescence homogeneously distributed in both hypha and conidia cells (Figures 5A,B). To assess the functionality of the new vectors in other *Trichoderma* species, we designed a plasmid carrying the *mCherry* encoding gene (pTrichoGate-4) under the control of the *gpdh1* (pTrichoGate-2) promoter and *T-nos* terminator (pTrichoGate-16). This vector was linearized and transformed into *T. sp. “atroviride B”* LU132 protoplasts. The expression of mCherry was analyzed by fluorescence microscopy (Supplementary Figure 5), indicating that these vectors are suitable for use in other *Trichoderma* species.

### Using Codon-Optimized Fluorescent Markers for Protein Localization Studies in *T. virens*

To test the effectiveness of the TrichoGate system to localize proteins in *Trichoderma*, we designed chimeric proteins between proteins from *T. virens* and fluorescent markers encoding in the TrichoGate plasmids. We choose Sm1, a well small-secreted protein that contains a signal peptide for secretion (Djonovic et al., 2006). The second protein chosen was the transcription factor TRIVIDRAFT_158742 (called here TvTF1), which contains a classical Nuclear Localization Signal in the N-terminal region (RTSKRRCIH)^6^. Localization vectors were created successfully using the TrichoGate strategy (Supplementary Figure 2). Sm1 was fuse to mCherry while TvTF1 to eGFP using a linker between the *Trichoderma* proteins and the fluorescent markers (Figure 5). Genetic transformation of *T. virens* protoplasts was performed using linearized localization vectors (Sm1 and TvTF1). Amplification of mCherry (730 bp) and eGFP (729 bp) ORF were used to confirm integration of the vector in the *T. virens* genome of the positive mutants (data not shown). Protein extractions of mycelia (Sm1-Loc and TvTF1-Loc mutants) and the supernatant fraction (Sm1-Loc mutants) were performed to verify protein localization and secretion. Western blot results showed that both fusions tagged proteins were present in the total protein extracts [Sm1-linker-mCherry fusion (∼41 kDa)] and [TvTF1-linker-eGFP (∼79 kDa)] from *T. virens* mycelia, and the chimeric protein Sm1-linker-mCherry was identified in the secreted fraction (Supplementary Figure 6). To visualize protein localization during fungal development, Sm1-linker-mCherry and TvTF1-linker-eGFP fusion proteins were expressed in *T. virens* independently, under the constitutive *gpdh* and *tef1*α promoter, respectively (Supplementary Figure 6). Fluorescence observations revealed that SM1 is expressed and localized in the cytosol or is attached to the fungal membrane (Figures 5C,D), and TvTF1 is localized in the nucleus (Figures 5E,F).

### Generation of a Novel Carboxin Resistant Marker in *T. virens*

#### Carboxin Sensitivity in *T. virens*

**FIGURE 6:**
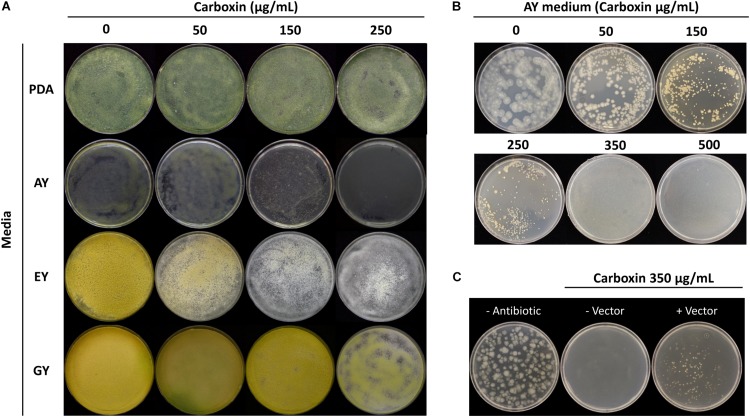
*Trichoderma virens* carboxin resistance. **(A)** Carboxin effect for mycelial growth of *T. virens* on different media [potato dextrose agar (PDA), acetate-acetic/yeast extract medium (AY), ethanol/yeast extract medium (EY) and glucose/yeast extract medium (GY)] containing various concentrations of carboxin. The wild-type (WT) strain was cultured for 7 days. **(B)** Carboxin sensitivity in *T. virens* WT on AY medium after 14 d.p.i. **(C)** Selection of carboxin resistance transformants of *T. virens*. Protoplasts were transformed with linearized pTrichoGate-19 vector, and then cultured on AY medium containing carboxin 350 μg/mL after 7 d.p.i.

The sensitivity of *T. virens* to carboxin was determined before the transformation. Four different media were evaluated to establish which medium was the most effective in synergy with carboxin to inhibit *T. virens* germination and growth. Carboxin sensitivity tested after 7 d.p.i on germination and growth of *T. virens* WT growing on PDA, EY and GY plates containing 0 to 250 μg/mL carboxin concentration, showed that *T. virens* germination and growth was not affected. In addition, the AY plates with the highest concentration of carboxin (250 μg/mL) resulted in *T. virens* germination inhibition (no geminated conidia or hyphae were detected) (Figure 6A). However, after 14 d.p.i small colonies were observed on AY medium at 250 μg/mL. Therefore, AY medium amended with 0–500 μg/mL carboxin was tested; no *T. virens* spore germination was observed from 350 to 500 μg/mL after 14 d.p.i (Figure 6B). Based on these results, AY medium amended with 350 μg/mL carboxin was used on for the isolation of carboxin-resistant mutants.

#### Design of Carboxin Resistance Marker for *T. virens*

The generation of the plasmid containing the carboxin resistance marker for *T. virens* was achieved using double-joint PCR to create a point mutation in the SdhBTv subunit (substituting His for Leu in codon 246, Supplementary Figure 3); additional point mutations were necessary to remove the endogenous *Bsa*I recognition sites without affecting the coding sequence. Three rounds of PCR were performed to generate the point mutations desired (Supplementary Figure 3A). By using standard molecular procedures, the entire CbxR cassette was amplified and subcloned into a pCR-Blunt backbone. The final product was sequenced and the point mutations verified (Supplementary Figure 3B) and called pTrichoGate-19. This construct was used for transform *T. virens* protoplasts as described above with 10 μg of the linearized pTrichoGate-19 vector (carboxin resistance cassette). Mutants emerged on selective medium 7 d.p.i and were transferred onto new AY selection plates amended with 350 μg carboxin/mL (Figure 6C). This demonstrated that pTrichoGate-19 was efficient in the generation of *T. virens* resistant mutants to carboxin.

## Discussion

The aim of this study was the design of a Golden Gate strategy for the analysis of functional genes in *T. virens*. As a result, the TrichoGate cloning system has provided a reliable, fast and effective method for the construction of different vectors (e.g., deletion, complementation, overexpression and localization) to study the genetics and biology of *Trichoderma*.

Linear DNA construction has been a time-limiting step that has been overcome throughout the implementation of the Golden Gate cloning system. Overall, Golden Gate is a simple, highly efficient and low-cost cloning strategy that speeds up the genetic manipulation of plants, fungi and bacteria by using only one type IIs restriction endonuclease for the assembly of multi genetic constructs, thus reducing the experimental steps in cloning processes (Lampropoulos et al., 2013; Andreou and Nakayama, 2018; Hernanz-Koers et al., 2018). For the design of the TrichoGate cloning system in *T. virens*, the commercially available endonuclease *Bsa*I (isoschizomer: *Eco*31I) was selected based on: (i) the generation of sticky ends after hydrolysis (with six bp separated by a single random nucleotide from the site of hydrolysis and generates a four nucleotide overhang) that permits efficient ligation reactions, and (ii) the restriction and the recognition sites which are not separated by more than ten bases, allowing its incorporation into primers of suitable length. Moreover, the design of specific inverse oriented *Bsa*I sites (on primers) allows the directional fusion of the flanking regions to receptor vectors (Terfrüchte et al., 2014). However, the use of other type IIs restriction enzymes is also suitable for the implementation of Golden Gate methodology (e.g., *Bbs*I or *Bsm*BI) (Sarrion-Perdigones et al., 2011; Housden and Perrimon, 2016).

In order to implement an efficient cloning system, all synthetic DNA molecules including promoters, markers, tags, terminators and resistance cassettes used through this technique were subcloned into the entry vectors for their use in the construction of multipurpose vectors for the study of *T. virens*. The design of modules containing genetic elements of interest has been previously implemented by Weber et al. (2011), demonstrating the reliability of the system to assemble complex constructs in a single digestion-ligation reaction, thus reducing the time of the construct generation (Engler et al., 2008). Similarly, the development of the GreenGate system highlights the use of modules including promoters, N- and C-terminal tags, coding sequence, terminator and resistance cassettes for the generation of plant transformation vectors (Lampropoulos et al., 2013).

### Generation of Time-Effective Knockout and Complementation Mutants in *T. virens*

The generation of deletion mutants in filamentous fungi is particularly challenging due to the low percentages of homologous recombination events. In *Trichoderma* spp. specifically, this percentage is approximately 2% (Mach and Zeilinger, 1998). However, in this work it was demonstrated that the generation of deletion constructs in *T. virens* using the TrichoGate cloning strategy coupled with *Agrobacterium*-mediated transformation that should be applicable for any *Trichoderma* species, resulted in an increase of homologous recombination events compared to conventional protoplast transformation methods, which generates a large number of transformants with ectopic integrations (Mach and Zeilinger, 1998). The results were consistent with previous reports in *T. atroviride*, where using *Agrobacterium*-mediated transformation increased the rate of homologous recombination (>60%) (Zeilinger, 2004).

The use of the Golden Gate cloning system to generate deletion constructs in fungi and bacteria as a strategy to overcome usual limitations of conventional methods has been implemented before in *U. maydis*, *A. nidulans* (Terfrüchte et al., 2014), *Penicillium digitatum* (Hernanz-Koers et al., 2018) and *Bacillus anthracis* (Wang et al., 2018). Moreover, the importance of flanking regions length when the Golden Gate cloning system is applied in filamentous fungi has been proved to be 1 kb for *U. maydis*, reaching 59% of homologous recombination events. In contrast, the use of shorter flanks led to increased rates of ectopic integrations (Terfrüchte et al., 2014). The results of the TrichoGate cloning system demonstrate that the homologous recombination event was higher than 50% when flanking regions from 1.0 to 1.3 kb were used for the generation of deletion mutants in *T. virens.* Furthermore, the construction of a complementation vector containing the geneticin resistance cassette was successfully implemented in *T. virens*, which should assist with functional analysis of targeted genes in *Trichoderma* that require efficient complementation vectors. The use of the geneticin resistance cassette as a selection marker for the genetic manipulation in *Trichoderma* spp. has been reported in the past for the complementation of a VELVET protein gene (*vel1*) (Mukherjee and Kenerley, 2010) and the generation of mutants of a constitutively activated version of the Gα subunit-encoding *tga3* gene (Gruber et al., 2012).

The Golden Gate cloning system has used to develop a multiplex CRISPR vectors for various downstream applications. This method was called ASAP-cloning (Adaptable System for Assembly of multiplexed Plasmid) (Zuckermann et al., 2018). The TrichoGate system can be modified for CRISPR applications by the integration of a self-replication fragment in the recipient plasmid; additionally, this plasmid can contain the U6 promoter, the gRNA scaffold and the terminator, although these components can be part of different entry vectors. By combining the TrichoGate and the system of subcloning reported by Zuckermann et al. (2018), is possible to integrate one or multiple gRNA expression cassettes using a PCR-on-ligation reaction (Zuckermann et al., 2018).

### Fluorescent Marker and Localization of *Trichoderma* Proteins

*Trichoderma* spp. closely interact with plants and other microorganisms (Harman et al., 2004). Microscopic observations of plant-fungi interactions have allowed the detection of specialized fungal structures that facilitate root colonization as well as fungal growth in both inter- and intracellular spaces (Nogueira-Lopez et al., 2018). In addition, mycoparasitic *Trichoderma* spp. utilize infective structures for the penetration of the cell wall of the host fungus (Guzman-Guzman et al., 2019). Therefore, the creation of fluorescent marker vectors (e.g., mCherry, eGFP and 3X eGFP) by using the TrichoGate cloning system may facilitate the monitoring of *Trichoderma* during the colonization of plant tissues and mycoparasitism. For example, the application of green fluorescent protein reporter systems during the study of three-way (*Trichoderma-*pathogen-plant) interactions (Lu et al., 2004) showed the effectiveness of the GFP marker for the *in situ* evaluation of *T. atroviride*-plant and/or *T. atroviride*-microbe interactions, including the results of codon optimization on mCherry fluorescent marker created in this study, showed an optimal visualization of *T. virens* and *T. atroviride* (mycelia and conidia).

Plant–microbe interactions are regulated by the secretion of several molecules that facilitate and maintain the interactions; among them, a variety of proteins that regulate the crosstalk between plants and microbes (Doehlemann and Hemetsberger, 2013). Therefore, the construction of protein-localization vectors in *Trichoderma* will assist in developing a deeper understanding of its interaction with plants. Localization vectors designed using the TrichoGate cloning system and the subsequent *T. virens* transformation enabled the localization of the Sm1-linker-mCherry and TvTF1-linker-eGFP recombinant proteins to corroborate the functionality of the fusion-protein linker. In addition, it was confirmed that SM1 was found in the secreted fraction under axenic conditions, which has been previously reported by Djonovic et al. (2006). Furthermore, Sm1 has been identified as part of the apoplastic secretome during the *T. virens*-maize interaction (Nogueira-Lopez et al., 2018). Moreover, TvTF1 [Zn2-Cys6 fungal transcription factor involved in secondary metabolism in *T. virens* (Nieto-Jacobo et al., in preparation)] was visualized in the nucleus (Figures 4E,F). The findings derived from this research show the potential of the use of the TrichoGate cloning system to successfully localize fungal proteins of interest to characterize *Trichoderma*-plant interactions.

### Carboxin Resistance in *T. virens*

Hygromycin resistance is one of the most common and reliable antibiotic markers used in *Trichoderma* spp. for the generation of mutants. However, the development of novel resistance markers is indispensable for the genetic study of filamentous fungi in order to enable serial gene deletions and re-transformations. Therefore, a novel resistance-based vector such as carboxin could be a viable alternative for genetic transformation in *Trichoderma*. The carboxin resistance marker has been widely used in other fungal species such as *U. maydis* (Broomfield and Hargreaves, 1992), *A. oryzae* (Shima et al., 2009), *Mycosphaerella graminicola* (Skinner et al., 1998) and *M. oryzae* (Guo et al., 2016). Carboxin inhibits the respiratory chain by altering the enzyme activity of SDH. The results showed that *T. virens* sensitivity to carboxin increased on acetate-containing media in comparison with the other media tested; acetate is the carbon source used as direct substrate in Krebs cycle, but this pathway is blocked via inhibition of the SDH activity by carboxin that may have lethal effect on fungi as previously reported in *A. oryzae* (Shima et al., 2009). Therefore, for the development of the transformation system, the AY medium was ideal for recovering *T. virens* carboxin-resistant mutants. Furthermore, the results showed that the point mutation (His to Leu) of the *sdhTv* gene that encodes the SDH iron-sulfur subunit of *T. virens* mutants conferred resistance to carboxin, suggesting a similar role in fungicide resistance as studied in other fungal models (Broomfield and Hargreaves, 1992; Skinner et al., 1998; Shima et al., 2009; Guo et al., 2016). Demonstrating that the carboxin resistance cassette may be suitable as a selection marker for the generation of mutants in *T. virens.*

## Conclusion

In conclusion, the present study demonstrates the functionality of a high-efficiency cloning system using the Golden Gate methodology applied to *Trichoderma* called TrichoGate. This system facilitates the generation of multipurpose vectors including localization of protein studies, tagging proteins for immune precipitation studies, gene deletion, overexpression of particular genes and gene complementations. Our system, together with gene editing using CRISPR/Cas9 technology in *Trichoderma* will be essential tools to conduct comprehensive studies of its biology.

## Data Availability Statement

The datasets generated for this study can be found in the GenBank MN007105–MN007125.

## Author Contributions

GN-L, AM-M, FP-A, SI, MN-J, and SL performed the experimental work. GN-L and AM-M designed the experiments. GN-L, AM-M, JS, FP-A, SI, SL, and MN-J discussed and interpreted the results. GN-L and AM-M designed the research. AM-M, JS, and AS contributed to the chemicals and scientific advice. GN-L, FP-A, and AM-M wrote the manuscript. All authors reviewed the final version of the manuscript.

## Conflict of Interest

JS was employed by Lincoln Agritech Ltd. The remaining authors declare that the research was conducted in the absence of any commercial or financial relationships that could be construed as a potential conflict of interest.

## Footnotes

1 https://genome.jgi.doe.gov/Trichoderma/Trichoderma.info.html

2 https://www.uniprot.org/proteomes/

3 http://www.kazusa.or.jp/codon/

4 https://www.addgene.org/

5 http://www.kazusa.or.jp/codon/

6 http://nls-mapper.iab.keio.ac.jp/cgi-bin/NLS_Mapper_y.cgi
