# Supplementary material for: TrichoGate: An Improved Vector System for a Large Scale of Functional Analysis of Trichoderma Genes
Source: Front Microbiol. 2019 Dec 10;10:2794. doi: 10.3389/fmicb.2019.02794 (PMC6915037; doi:10.3389/fmicb.2019.02794)
Supplement: Supplementary file 1 [file Data_Sheet_1.docx]

TrichoGate: An improved Vector System for a Large Scale of Functional Analysis of *Trichoderma*  Genes

Guillermo Nogueria-Lopez^1^, Fabiola Padilla-Arizmendi^1^, Sarah Inwood^1,2^, Sarah Lyne^1^, Johanna M Steyaert ^1,3^, Maria Fernanda Nieto-Jacobo^1,4^, Alison Stewart^1,5^, Artemio Mendoza-Mendoza^1,*^

^1^ Bio-Protection Research Centre, Lincoln University, Lincoln 7647, New Zealand

^2^ Biochemistry Department, University of Otago, Dunedin, New Zealand

^3^ Lincoln Agritech Ltd, PO Box 69133, Lincoln, Christchurch 7460, New Zealand

^4^ Plant & Food Research Gerald St, Lincoln 7608, New Zealand

**^5^**  Foundation For Arable Research (FAR), Templeton, Christchurch 7678, New Zealand

*** Corresponding Author:**Artemio Mendoza-Mendoza
Artemio.mendoza@lincoln.ac.nz

Supplementary Material

# Protoplast transformation

Transformation of *T. virens* protoplasts with the vectors created was performed according to the method reported by Baek and Kenerley (1998) with modifications for the protoplast isolation described by Lawry (2016). *T. virens* conidia (1x10^5^ conidia/mL) contained in 100 μL of nanopure water were spread onto a large potato dextrose agar (PDA) (Difco, USA) petri dish (150 mm x 150 mm) (NEST Scientific, USA) which was previously covered with sterile cellophane sheet. Plates were incubated for 16 h at 25°C in darkness. For digestion of *T. virens* mycelia a digestion solution was prepared containing 0.5 g Glucanex (Novozyme, Denmark) dissolved in 50 mL of 0.7 M sterile mannitol osmoticum solution, pH 5.5, containing 50 mM CaCl2, 0.7 M mannitol and 50 mM MES hydrate. The enzymatic solution was sterilized using 0.45 μm cellulose acetate membrane syringe filters (GVS Filter Technology, UK). The cellophane covered with *T. virens* mycelia was moved into a new large petri dish and then 15 mL of digestion solution were placed on the cellophane until it was totally immersed in the solution. The plate containing the digestion mix was incubated for 4 h at 25°C and continuous shaking at 150 rpm. Digested mycelia were filtered twice to recover *T. virens* protoplasts, firstly through two layers of sterile Miracloth and secondly through a Swinnex filter holder (Merck Millipore, USA) using 40 μm nylon mesh. The protoplast solution was centrifuged at 4,000 rpm for 10 min at 4°C and the supernatant was removed by inversion. The protoplast pellet was washed with 20 mL of 0.7 M mannitol osmoticum solution and centrifuged at 7,000 rpm for 7 min and then suspended in 500 μL of 0.7 M mannitol osmoticum solution. Protoplast concentration was determined using a haemocytometer.

For protoplast transformation, ten micrograms of DNA construct were linearized using the following conditions. The digested vector was purified and visualised as previous described to verify their integrity. The linearized constructs (10 μg) were added to 240 μL containing 10^8^ protoplasts in mannitol osmoticum solution and mixed gently. The mix protoplast + DNA construct was incubated on ice for 20 min. After incubation, 130 μL of 40% polyethylene glycol (PEG) solution (40 g PEG 4000, dissolved in 100 mL of 0.7 mannitol osmoticum) were added and mixed by inversion; this step was repeated 2 times and then incubated for 20 min at RT. For regeneration of transformants, the protoplast solution was mixed with 10 mL recovery media (RM) (PDB; 1% bacteriological agar; 0.5 M sucrose) + 100 μg/mL hygromycin B (AG Scientific, USA). The mixture was then poured onto prepared plates containing 10 mL RM + 100 μg/mL hygromycin B. A negative control containing RM + hygromycin B and protoplast solution without DNA construct was used, together with a positive control consisting of RM and protoplast solution. Plates were incubated for 7 d at 25°C.

# Genomic DNA isolation of *T. virens*

Isolation of *T. virens* DNA from mycelia was performed using the following methodology: in 100 mL sterile flasks, *T. virens* conidia (1x106) were grown in 30 mL of sterile PDB media for 36 h at 25°C with continuous shaking at 150 rpm. Mycelia were harvested on two layers of Miracloth and rinsed with sterile nanopure water. Collected mycelia were ground into a fine powder in liquid nitrogen to disrupt the cells. Mycelia powder (100 mg) was poured into a 2 mL screw-capped microcentrifuge tube and homogenised in 0.5 mL of lysis buffer (2% Triton X; 1% SDS; 100 mM NaCl; 10 mM Tris-HCl pH 8; 1 mM EDTA) and 0.5 mL phenol:chloroform:isoamyl alcohol (25:24:1). Samples were vortexed for 10 s and centrifuged at 14,500 x g for 10 min at 4°C. The supernatant was collected and transferred into a new 2 mL centrifuge tube, and then 2.5 volumes of absolute cold ethanol were added. The samples were mixed carefully to avoid damaging DNA and centrifuged at 14,500 x g for 5 min at 4°C. The supernatant was removed carefully without disturbing the DNA pellet and then centrifuged for 2 min. The remaining ethanol was removed with a pipette, and the pellet was re-suspended in 30 μL ultrapure DNase/RNase distilled water and incubated for 24 h at 4°C. The DNA from the samples was quantified using a Qubit dsDNA HS assay kit (Thermo Fisher Scientific, USA).

# PCR reactions that were carried out for mutants´ confirmation are described below

1. **Hygromycin Resistance Cassette (HRC) Amplification:** HRC amplification was carried out in a final volume of 20 µL containing 0.5 µL of genomic DNA, 12.5 µL of Phusion High Fidelity PCR Master Mix with HF buffer (Thermo Fisher Scientific) and 1 µL of each primer (LU357 and LU358). The PCR cycling conditions were as follows: denaturation 98 °C 1 min; 35 cycles at 98 °C 15 s, 60 °C 15 s and 72 °C 1 min; and a final extension at 72 °C 10 min.
2. **External left frank to the HRC amplification (TS-Ext LF to HRC):** TS-Ext LF to HRC amplification was carried out in a final volume of 50 µL. PCR mix was prepared as described for the amplification of the full construct. The primers used for this reaction were LU217 and TS1-Ext LF for knockout of *ts1* or LU217 and TS2-Ext LF for knockout of *ts2*. The PCR cycling conditions were the same as the used for hygromycin resistance gene amplification.
3. **HRC to external right flank amplification (HRC to Ext-RF).** HRC to Ext-RF amplification, was carried out in a final volume of 50 µL. The primers used for this reaction were LU218 and TS1-Ext RF for knockout of *ts1* and LU218 /TS2-Ext RF for knockout of *ts2.* PCR cycling conditions were the same described for HRC amplification.

All PCR reactions were done using a SuperCycler SC300, Kyratec; PCR products of all reactions were analyzed by electrophoresis using a 0.8% Agarose-TAE (HyAgarose, HydraGene) gel for 60 min at 100 V; 5 µL of each PCR reaction was used (Supplementary Figure 4).

# References

Baek, J.M., and Kenerley, C.M. (1998). The arg2 gene of *Trichoderma virens*: cloning and development of a homologous transformation system. *Fungal Genetics Biology* 23(1)**,** 34-44. doi: 10.1006/fgbi.1997.1025.

Lawry, R. (2016). *Cross-communication between Trichoderma and plants during root cololnisation.* PhD, Lincoln University.

## Supplementary Data

## Codon optimization in *Trichoderma*

Sequences of codon-optimized markers (mCherry, eGFP and 3X eGFP) and tags (3X c-Myc, 3X FLAG and 3X HA) for *Trichoderma.* The nucleotides in red indicate codon optimization*.*

### mCherry codon-optimized

| Optimized | 13 | ATGGTCAGCAAGGGCGAAGAAGACAACATGGCTATTATCAAGGAGTTTATGCGATTCAAG |
| --- | --- | --- |
| Original | 13 | ATGGTGAGCAAGGGCGAGGAGGATAACATGGCCATCATCAAGGAGTTCATGCGCTTCAAG |
|  |  |  |
| Optimized | 73 | GTTCACATGGAAGGCAGCGTCAACGGCCACGAGTTCGAAATCGAGGGTGAAGGCGAGGGC |
| Original | 73 | GTGCACATGGAGGGCTCCGTGAACGGCCACGAGTTCGAGATCGAGGGCGAGGGCGAGGGC |
|  |  |  |
| Optimized | 133 | CGACCCTACGAGGGCACCCAGACTGCCAAGCTCAAGGTCACCAAGGGCGGTCCCCTCCCT |
| Original | 133 | CGCCCCTACGAGGGCACCCAGACCGCCAAGCTGAAGGTGACCAAGGGTGGCCCCCTGCCC |
|  |  |  |
| Optimized | 193 | TTCGCTTGGGACATCCTGTCTCCACAGTTCATGTACGGTAGCAAGGCCTATGTTAAGCAT |
| Original | 193 | TTCGCCTGGGACATCCTGTCCCCTCAGTTCATGTACGGCTCCAAGGCCTACGTGAAGCAC |
|  |  |  |
| Optimized | 253 | CCAGCTGACATTCCCGATTACCTCAAGCTGTCCTTCCCTGAAGGCTTTAAGTGGGAGCGT |
| Original | 253 | CCCGCCGACATCCCCGACTACTTGAAGCTGTCCTTCCCCGAGGGCTTCAAGTGGGAGCGC |
|  |  |  |
| Optimized | 313 | GTCATGAACTTTGAAGATGGCGGTGTCGTTACCGTTACTCAGGACTCTAGCCTTCAAGAT |
| Original | 313 | GTGATGAACTTCGAGGACGGCGGCGTGGTGACCGTGACCCAGGACTCCTCCCTGCAGGAC |
|  |  |  |
| Optimized | 373 | GGCGAGTTCATCTACAAGGTCAAGCTGCGCGGCACCAACTTTCCTTCAGACGGCCCAGTT |
| Original | 373 | GGCGAGTTCATCTACAAGGTGAAGCTGCGCGGCACCAACTTCCCCTCCGACGGCCCCGTA |
|  |  |  |
| Optimized | 433 | ATGCAGAAGAAGACTATGGGCTGGGAAGCCTCCTCAGAGCGCATGTATCCTGAAGATGGT |
| Original | 433 | ATGCAGAAGAAGACGATGGGCTGGGAGGCCTCCTCCGAGCGGATGTACCCCGAGGACGGC |
|  |  |  |
| Optimized | 493 | GCTCTGAAGGGCGAGATTAAGCAGCGACTTAAGTTGAAGGACGGCGGTCACTACGATGCC |
| Original | 493 | GCCCTGAAGGGCGAGATCAAGCAGAGGCTGAAGCTGAAGGACGGCGGCCACTACGACGCT |
|  |  |  |
| Optimized | 553 | GAGGTCAAGACCACTTATAAGGCTAAGAAGCCCGTCCAACTTCCTGGCGCCTACAACGTC |
| Original | 553 | GAGGTCAAGACCACCTACAAGGCCAAGAAGCCCGTGCAGCTGCCCGGCGCCTACAACGTC |
|  |  |  |
| Optimized | 613 | AACATCAAGTTGGACATTACCTCCCATAACGAAGATTATACTATTGTTGAGCAATATGAG |
| Original | 613 | AACATCAAGTTGGACATCACCTCCCACAACGAGGACTACACCATCGTGGAACAGTACGAA |
|  |  |  |
| Optimized | 673 | CGAGCCGAGGGCCGACACAGCACTGGTGGTATGGACGAACTCTACAAGTAA |
| Original | 673 | CGCGCCGAGGGCCGCCACTCCACCGGCGGCATGGACGAGCTGTACAAGTAA |

### eGFP codon-optimized

| Optimized | 13 | ATGGCTACTATGGTCAGCAAGGGCGAAGAACTTTTTACTGGTGTCGTCCCAATCCTGGTC | | | | | | |  |
| --- | --- | --- | --- | --- | --- | --- | --- | --- | --- |
| Original | 13 | ATGGCCACCATGGTGAGCAAGGGCGAGGAGCTGTTCACCGGGGTGGTGCCCATCCTGGTC | | | | | | |  |
| Optimized | 73 | GAACTGGATGGCGATGTCAACGGTCATAAGTTCTCTGTTAGCGGCGAGGGTGAAGGCGAC | | | | | | |  |
| Original | 73 | GAGCTGGACGGCGACGTAAACGGCCACAAGTTCAGCGTGTCCGGCGAGGGCGAGGGCGAT | | | | | | |  |
| Optimized | 133 | GCTACCTACGGCAAGCTCACTTTGAAGTTTATCTGCACCACTGGCAAGCTCCCCGTCCCT | | | | | | |  |
| Original | 133 | GCCACCTACGGCAAGCTGACCCTGAAGTTCATCTGCACCACCGGCAAGCTGCCCGTGCCC | | | | | | |  |
| Optimized | 193 | TGGCCAACCCTCGTTACCACTCTGACTTACGGCGTTCAGTGCTTCAGCCGCTATCCAGAC | | | | | | |  |
| Original | 193 | TGGCCCACCCTCGTGACCACCCTGACCTACGGCGTGCAGTGCTTCAGCCGCTACCCCGAC | | | | | | |  |
| Optimized | 253 | CACATGAAGCAACATGATTTCTTTAAGTCCGCCATGCCCGAGGGTTACGTCCAGGAACGC | | | | | | |  |
| Original | 253 | CACATGAAGCAGCACGACTTCTTCAAGTCCGCCATGCCCGAAGGCTACGTCCAGGAGCGC | | | | | | |  |
| Optimized | 313 | ACCATCTTCTTTAAGGACGATGGCAACTATAAGACCCGAGCTGAGGTCAAGTTCGAAGGT | | | | | | |  |
| Original | 313 | ACCATCTTCTTCAAGGACGACGGCAACTACAAGACCCGCGCCGAGGTGAAGTTCGAGGGC | | | | | | |  |
| Optimized | 373 | GACACTCTCGTTAACCGTATCGAGCTGAAGGGCATTGACTTTAAGGAAGATGGTAACATT | | | | | | |  |
| Original | 373 | GACACCCTGGTGAACCGCATCGAGCTGAAGGGCATCGACTTCAAGGAGGACGGCAACATC | | | | | | |  |
| Optimized | 433 | CTTGGCCACAAGTTGGAGTACAACTATAACTCACATAACGTCTACATCATGGCCGATAAG | | | | | | |  |
| Original | 433 | CTGGGGCACAAGCTGGAGTACAACTACAACAGCCACAACGTCTATATCATGGCCGACAAG | | | | | | |  |
| Optimized | 493 | CAGAAGAACGGTATTAAGGTTAACTTCAAGATCCGCCACAACATTGAAGACGGCTCCGTC | | | | | | |  |
| Original | 493 | CAGAAGAACGGCATCAAGGTGAACTTCAAGATCCGCCACAACATCGAGGACGGCAGCGTG | | | | | | |  |
| Optimized | 553 | CAACTGGCTGATCATTACCAGCAAAACACCCCTATTGGTGACGGCCCCGTTCTCCTGCCT | | | | | | |  |
| Original | 553 | CAGCTCGCCGACCACTACCAGCAGAACACCCCCATCGGCGACGGCCCCGTGCTGCTGCCC | | | | | | |  |
| Optimized | 613 | GATAACCACTATCTTTCCACTCAGTCAGCTTTGTCTAAGGACCCAAACGAGAAGCGAGAT | | | | | | |  |
| Original | 613 | GACAACCACTACCTGAGCACCCAGTCCGCCCTGAGCAAAGACCCCAACGAGAAGCGCGAT | | | | | | |  |
| Optimized | 673 | CACATGGTCCTGCTGGAGTTTGTCACTGCTGCTGGTATCACCCTGGGCATGGACGAGTTG | | | | | | |  |
| Original | 673 | CACATGGTCCTGCTGGAGTTCGTGACCGCCGCCGGGATCACTCTCGGCATGGACGAGCTG | | | | | | |  |
| Optimized | 733 | TATAAGTAA |  |  |  |  |  |  |  |
| Original | 733 | TACAAGTAA |  |  |  |  |  |  |  |

### 3X eGFP codon-optimized

| Optimized | 13 | ATGGTCTCAAAGGGCGAAGAGCTGTTCACTGGTGTCGTTCCCATCCTGGTCGAATTGGAC |
| --- | --- | --- |
| Original | 13 | ATGGTGAGCAAGGGCGAGGAGCTGTTCACCGGGGTGGTGCCCATCCTGGTCGAGCTGGAC |
|  |  |  |
| Optimized | 73 | GGCGATGTCAACGGTCATAAGTTCTCCGTTAGCGGCGAGGGTGAAGGCGACGCTACCTAC |
| Original | 73 | GGCGACGTAAACGGCCACAAGTTCAGCGTGTCCGGCGAGGGCGAGGGCGATGCCACCTAC |
|  |  |  |
| Optimized | 133 | GGCAAGCTCACCTTGAAGTTCATCTGCACCACTGGCAAGCTCCCCGTCCCTTGGCCAACC |
| Original | 133 | GGCAAGCTGACCCTGAAGTTCATCTGCACCACCGGCAAGCTGCCCGTGCCCTGGCCCACC |
|  |  |  |
| Optimized | 193 | CTCGTTACCACTCTGACTTACGGCGTTCAGTGCTTTTCTCGCTACCCCGACCACATGAAG |
| Original | 193 | CTCGTGACCACCCTGACCTACGGCGTGCAGTGCTTCAGCCGCTACCCCGACCACATGAAG |
|  |  |  |
| Optimized | 253 | CAACATGATTTCTTTAAGTCCGCCATGCCTGAGGGTTACGTCCAGGAACGCACCATCTTC |
| Original | 253 | CAGCACGACTTCTTCAAGTCCGCCATGCCCGAAGGCTACGTCCAGGAGCGCACCATCTTC |
|  |  |  |
| Optimized | 313 | TTTAAGGACGATGGCAACTATAAGACCCGAGCTGAGGTCAAGTTCGAAGGTGACACCCTC |
| Original | 313 | TTCAAGGACGACGGCAACTACAAGACCCGCGCCGAGGTGAAGTTCGAGGGCGACACCCTG |
|  |  |  |
| Optimized | 373 | GTCAACCGTATCGAGCTGAAGGGCATTGACTTTAAGGAAGATGGTAACATTCTTGGCCAC |
| Original | 373 | GTGAACCGCATCGAGCTGAAGGGCATCGACTTCAAGGAGGACGGCAACATCCTGGGGCAC |
|  |  |  |
| Optimized | 433 | AAGTTGGAGTACAACTATAACTCACATAACGTCTACATCATGGCCGATAAGCAGAAGAAC |
| Original | 433 | AAGCTGGAGTACAACTACAACAGCCACAACGTCTATATCATGGCCGACAAGCAGAAGAAC |
|  |  |  |
| Optimized | 493 | GGCATTAAGGTCAACTTCAAGATCCGCCACAACATTGAGGACGGCTCCGTCCAACTGGCT |
| Original | 493 | GGCATCAAGGTGAACTTCAAGATCCGCCACAACATCGAGGACGGCAGCGTGCAGCTCGCC |
|  |  |  |
| Optimized | 553 | GATCATTACCAGCAAAACACCCCTATCGGTGACGGCCCTGTCCTCCTGCCTGATAACCAC |
| Original | 553 | GACCACTACCAGCAGAACACCCCCATCGGCGACGGCCCCGTGCTGCTGCCCGACAACCAC |
|  |  |  |
| Optimized | 613 | TATCTTTCTACTCAGAGCGCCTTGTCCAAGGACCCCAACGAGAAGCGAGATCACATGGTC |
| Original | 613 | TACCTGAGCACCCAGTCCGCCCTGAGCAAAGACCCCAACGAGAAGCGCGATCACATGGTC |
|  |  |  |
| Optimized | 673 | CTCCTCGAGTTCGTTACCGCCGCTGGTATCACTCTCGGCATGGACGAGCTGTACAAGGTT |
| Original | 673 | CTGCTGGAGTTCGTGACCGCCGCCGGGATCACTCTCGGCATGGACGAGCTGTACAAGGTG |
|  |  |  |
| Optimized | 733 | TCTAAGGGCGAGGAACTCTTCACCGGCGTCGTTCCTATTCTCGTCGAACTGGACGGCGAC |
| Original | 733 | AGCAAGGGCGAGGAGCTGTTCACCGGGGTGGTGCCCATCCTGGTCGAGCTGGACGGCGAC |
|  |  |  |
| Optimized | 793 | GTCAACGGCCACAAGTTTTCTGTCAGCGGCGAGGGCGAGGGCGACGCCACCTACGGCAAG |
| Original | 793 | GTAAACGGCCACAAGTTCAGCGTGTCCGGCGAGGGCGAGGGCGATGCCACCTACGGCAAG |
|  |  |  |
| Optimized | 853 | CTCACCCTGAAGTTCATTTGCACCACTGGCAAGCTGCCCGTCCCTTGGCCTACCCTCGTT |
| Original | 853 | CTGACCCTGAAGTTCATCTGCACCACCGGCAAGCTGCCCGTGCCCTGGCCCACCCTCGTG |
|  |  |  |
| Optimized | 913 | ACTACTCTGACTTACGGCGTCCAGTGCTTCTCACGCTATCCAGATCACATGAAGCAGCAT |
| Original | 913 | ACCACCCTGACCTACGGCGTGCAGTGCTTCAGCCGCTACCCCGACCACATGAAGCAGCAC |
|  |  |  |
| Optimized | 973 | GATTTCTTTAAGTCTGCCATGCCCGAGGGCTATGTTCAAGAGCGTACTATCTTCTTTAAG |
| Original | 973 | GACTTCTTCAAGTCCGCCATGCCCGAAGGCTACGTCCAGGAGCGCACCATCTTCTTCAAG |
|  |  |  |
| Optimized | 1033 | GATGACGGTAACTACAAGACCCGAGCTGAAGTTAAGTTTGAGGGTGACACCCTCGTCAAC |
| Original | 1033 | GACGACGGCAACTACAAGACCCGCGCCGAGGTGAAGTTCGAGGGCGACACCCTGGTGAAC |
|  |  |  |
| Optimized | 1093 | AGAATCGAGCTTAAGGGTATTGACTTCAAGGAGGACGGCAACATCCTCGGTCATAAGTTG |
| Original | 1093 | CGCATCGAGCTGAAGGGCATCGACTTCAAGGAGGACGGCAACATCCTGGGGCACAAGCTG |
|  |  |  |
| Optimized | 1153 | GAATACAACTATAACTCTCATAACGTTTATATTATGGCTGACAAACAGAAGAACGGCATT |
| Original | 1153 | GAGTACAACTACAACAGCCACAACGTCTATATCATGGCCGACAAGCAGAAGAACGGCATC |
|  |  |  |
| Optimized | 1213 | AAGGTCAACTTTAAGATCCGTCATAACATCGAGGACGGCAGCGTTCAACTCGCCGACCAC |
| Original | 1213 | AAGGTGAACTTCAAGATCCGCCACAACATCGAGGACGGCAGCGTGCAGCTCGCCGACCAC |
|  |  |  |
| Optimized | 1273 | TATCAACAGAACACCCCTATCGGCGACGGTCCAGTCCTCCTGCCCGATAACCACTATCTC |
| Original | 1273 | TACCAGCAGAACACCCCCATCGGCGACGGCCCCGTGCTGCTGCCCGACAACCACTACCTG |
|  |  |  |
| Optimized | 1333 | TCAACTCAGTCTGCCCTGTCTAAGGACCCCAATGAGAAGCGCGACCACATGGTTCTCCTC |
| Original | 1333 | AGCACCCAGTCCGCCCTGAGCAAAGACCCCAACGAGAAGCGCGATCACATGGTCCTGCTG |
|  |  |  |
| Optimized | 1393 | GAGTTCGTCACCGCCGCTGGTATTACCCTTGGCATGGACGAGTTGTACAAGGTTAGCAAG |
| Original | 1393 | GAGTTCGTGACCGCCGCCGGGATCACTCTCGGCATGGACGAGCTGTACAAGGTGAGCAAG |
|  |  |  |
| Optimized | 1453 | GGCGAGGAACTTTTTACCGGCGTCGTTCCCATTCTTGTCGAGTTGGATGGCGACGTCAAC |
| Original | 1453 | GGCGAGGAGCTGTTCACCGGGGTGGTGCCCATCCTGGTCGAGCTGGACGGCGACGTAAAC |
|  |  |  |
| Optimized | 1513 | GGTCACAAGTTCTCCGTCTCAGGCGAGGGTGAAGGCGATGCAACCTACGGCAAGCTCACC |
| Original | 1513 | GGCCACAAGTTCAGCGTGTCCGGCGAGGGCGAGGGCGATGCCACCTACGGCAAGCTGACC |
|  |  |  |
| Optimized | 1573 | CTAAAGTTTATTTGCACTACCGGCAAGCTGCCCGTCCCTTGGCCGACCCTCGTTACAACT |
| Original | 1573 | CTGAAGTTCATCTGCACCACCGGCAAGCTGCCCGTGCCCTGGCCCACCCTCGTGACCACC |
|  |  |  |
| Optimized | 1633 | CTGACTTACGGCGTACAGTGCTTCAGCCGCTATCCTGATCACATGAAGCAGCACGATTTC |
| Original | 1633 | CTGACCTACGGCGTGCAGTGCTTCAGCCGCTACCCCGACCACATGAAGCAGCACGACTTC |
|  |  |  |
| Optimized | 1693 | TTTAAGAGCGCTATGCCAGAGGGCTATGTTCAAGAACGTACCATCTTCTTTAAGGATGAT |
| Original | 1693 | TTCAAGTCCGCCATGCCCGAAGGCTACGTCCAGGAGCGCACCATCTTCTTCAAGGACGAC |
|  |  |  |
| Optimized | 1753 | GGTAATTACAAAACCCGAGCTGAAGTCAAGTTTGAAGGTGACACCCTCGTCAACAGGATT |
| Original | 1753 | GGCAACTACAAGACCCGCGCCGAGGTGAAGTTCGAGGGCGACACCCTGGTGAACCGCATC |
|  |  |  |
| Optimized | 1813 | GAGCTGAAGGGTATTGACTTTAAGGAGGACGGCAACATTCTCGGTCATAAGTTGGAGTAT |
| Original | 1813 | GAGCTGAAGGGCATCGACTTCAAGGAGGACGGCAACATCCTGGGGCACAAGCTGGAGTAC |
|  |  |  |
| Optimized | 1873 | AACTACAACTCCCACAACGTTTATATTATGGCCGACAAGCAGAAGAACGGCATTAAGGTC |
| Original | 1873 | AACTACAACAGCCACAACGTCTATATCATGGCCGACAAGCAGAAGAACGGCATCAAGGTG |
|  |  |  |
| Optimized | 1933 | AACTTCAAAATCCGACATAACATCGAAGACGGCTCTGTCCAACTCGCCGACCACTACCAA |
| Original | 1933 | AACTTCAAGATCCGCCACAACATCGAGGACGGCAGCGTGCAGCTCGCCGACCACTACCAG |
|  |  |  |
| Optimized | 1993 | CAAAACACTCCCATCGGTGACGGCCCTGTTCTCCTGCCAGATAACCACTATCTGTCCACT |
| Original | 1993 | CAGAACACCCCCATCGGCGACGGCCCCGTGCTGCTGCCCGACAACCACTACCTGAGCACC |
|  |  |  |
| Optimized | 2053 | CAGTCAGCTCTTTCTAAGGACCCCAACGAGAAGCGTGATCACATGGTCCTCCTTGAGTTT |
| Original | 2053 | CAGTCCGCCCTGAGCAAAGACCCCAACGAGAAGCGCGATCACATGGTCCTGCTGGAGTTC |
|  |  |  |
| Optimized | 2113 | GTCACCGCTGCTGGTATCACCTTGGGTATGGACGAGTTGTATAAGTAA |
| Original | 2113 | GTGACCGCCGCCGGGATCACTCTCGGCATGGACGAGCTGTACAAGTAA |

### c-Myc codon-optimized

| Optimized | 20 | GAGCAGAAGCTCATCTCCGAGGAAGACCTGGCCGGCGCTGGTTCAGAACAAAAGCTCATC |
| --- | --- | --- |
| Original | 20 | GAGCAGAAGCTCATCTCGGAAGAGGACCTCGCCGGTGCCGGCTCGGAGCAGAAGCTCATC |
| Optimized | 80 | TCTGAGGAAGATTTGAGCGCCGGCGGTGCTGAGCAGAAGCTGATCAGCGAGGAAGACCTC |
| Original | 80 | TCGGAAGAGGACCTCTCGGCCGGTGGTGCCGAGCAGAAGCTCATCTCGGAAGAGGACCTC |

### FLAG codon-optimized

| Optimized | 20 | GACTACAAGGACGATGACGATAAGATCGCCGGCGCTGGTTCTGATTATAAGGATGACGAC | |
| --- | --- | --- | --- |
| Original | 20 | GACTACAAGGACGACGACGACAAGATCGCCGGTGCCGGCTCGGACTACAAGGACGACGAC | |
| Optimized | 80 | GATAAGATTAGCGCCGGCGGTGCTGATTATAAGGATGATGATGATAAGATC |  |
| Original | 80 | GACAAGATCTCGGCCGGTGGTGCCGACTACAAGGACGACGACGACAAGATC |  |

### HA codon-optimized

| Optimized | 20 | TACCCCTATGACGTCCCTGATTACGCTGGTGCTGCTTACCCCTACGACGTTCCCGATTAT | | | |
| --- | --- | --- | --- | --- | --- |
| Original | 20 | TACCCCTACGACGTGCCCGACTATGCCGGTGCCGCCTACCCCTACGACGTGCCCGATTAC | | | |
| Optimized | 80 | TCTGCTGGCGGTTACCCTTATGACGTCCCAGATTAC |  |  |  |
| Original | 80 | TCGGCTGGTGGTTACCCCTACGACGTGCCCGACTAT |  |  |  |

# Supplementary Figures

## Supplementary Figures


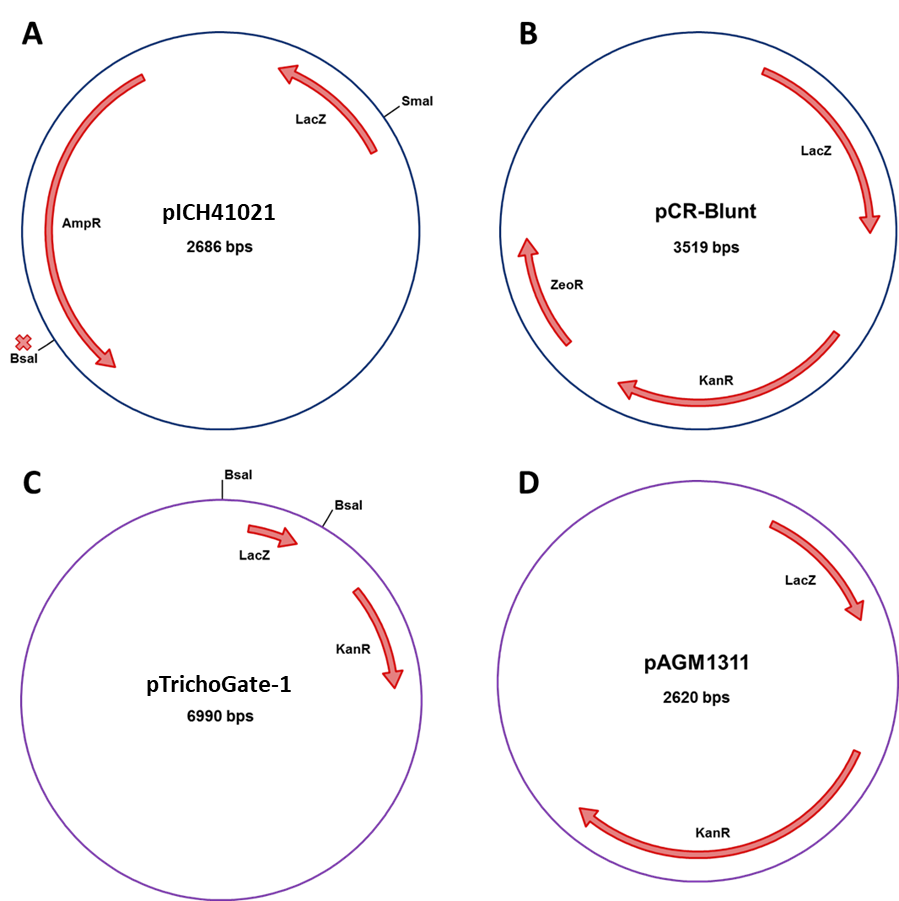


**Supplementary Figure 1.** **Cloning** **vectors used for the implementation of the TrichoGate cloning strategy.** Physical maps of entry vectors **(A)** pICH41021and **(B)** pCR-Blunt. Receptor vectors **(C)** pTrichoGate-and **(D)** pAGM1311.

**
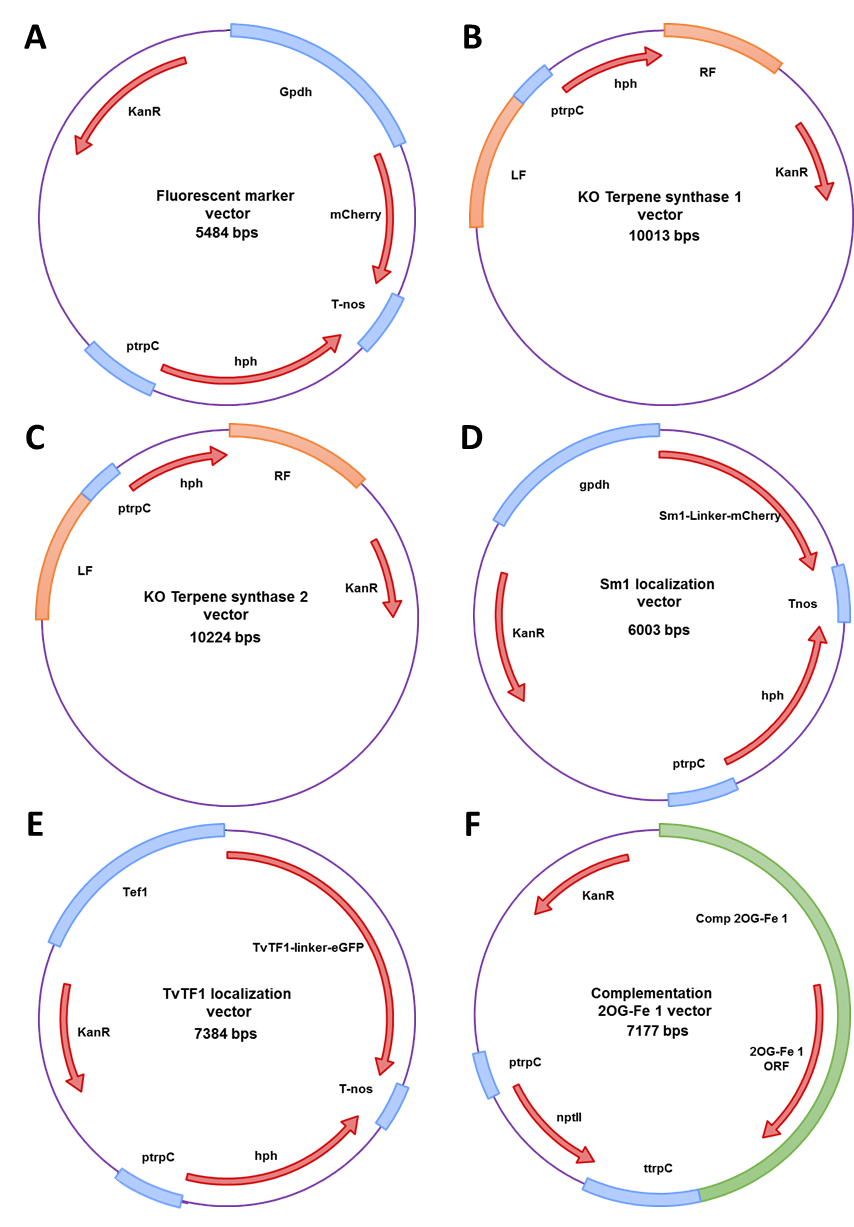
**

**Supplementary Figure 2.** **Scheme of vectors created using TrichoGate cloning strategy. (A)** Fluorescent marker vector (Gpdh+mCherry+T-nos+HygR). **(B)** Deletion TS1 vector (LF+HygR+RF). **(C)** Deletion TS2 vector (LF+HygR+RF). **(D)** Sm1 localization vector (Gpdh+Sm1-linker-mCherry+T-nos+HygR). **(E)** TvTF1 localization vector (Tef1+TvTF1-linker-eGFP+T-nos+HygR) and **(F)** Complementation TV2OG1 vector (Comp 2OG-Fe1+GtR).


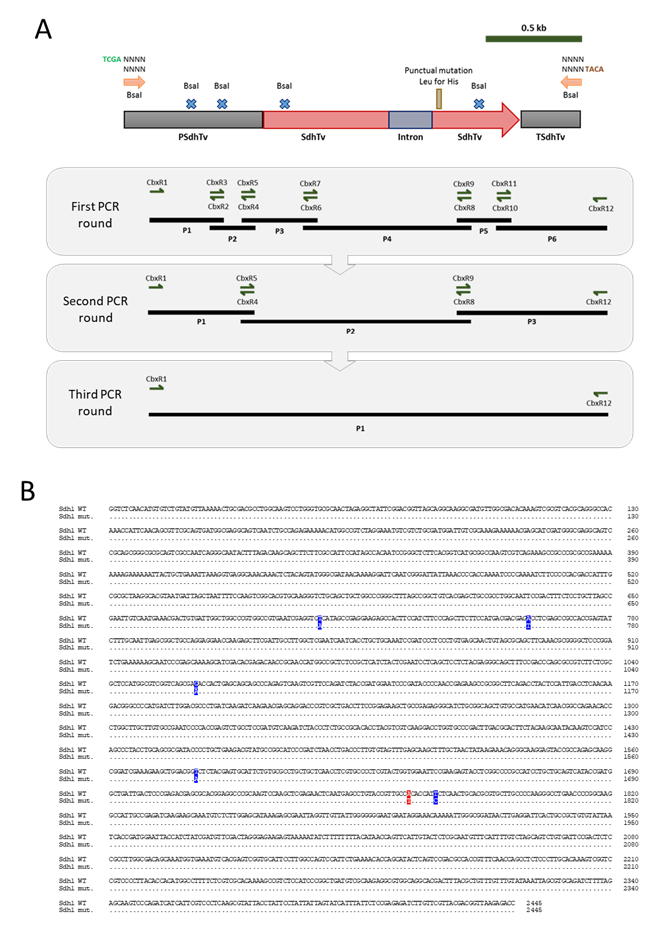


**Supplementary Figure 3.** **Strategy for the creation of carboxin resistance cassette in *T. virens*. (A)** Point mutations to eliminate *Bsa*I internal sites and confer resistance to carboxin on succinate dehydrogenase iron-sulphur subunit protein in *T. virens*. The three rounds of PCR and the primers used in each case are indicated in the grey rectangles. The black lines represent the PCR product generated by the primers combination used. The arrows next to the primers represent the direction of the primers. The sequence of primers are in Supplementary Table 1. **(B)** Comparison of the nucleotide sequence of *sdh1* wild type (Sdh1 WT) from *T. virens* and *sdh1* mutated (Sdh1 mut.). Identical nucleotides between both sequences are indicated by dots. The nucleotides labelled in blue represent the point mutations to eliminate the *Bsa*I sites. The nucleotides indicated in red represent the point mutation to increase the resistance to carboxin. This mutation changes the amino acids from His (CAC) to Leu (CTC) in codon 246.


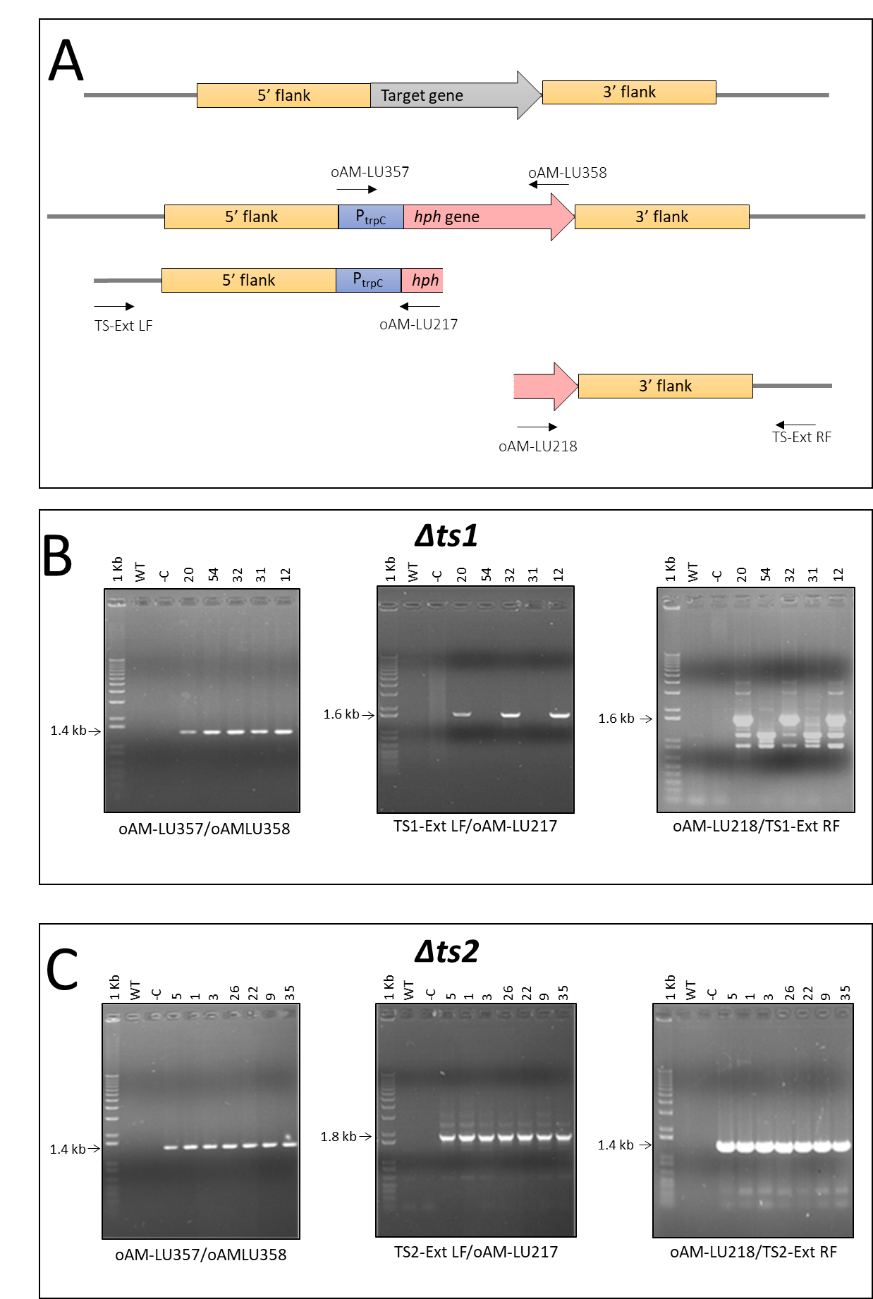


**Supplementary Figure 4.** **Terpene synthases deletion confirmation by PCR. (A)** Cartoon representation of the terpene synthase genes. The middle cartoon represents the double recombination where the putative *ts* gene is substituted by *hph*, the hygromycin resistant gene under control of promoter *Trpc*. Set of primers used to corroborate deletion of the ts mutants, insertion of the HygR cassette and homologous recombination. **(B)** Corroboration of double recombination in *ts1* deletion mutants. For the amplification of *hph* the primer combination oAM-LU357/oAM-LU358 was used; for the 5’ flank double recombination was generated by using the primer mixture TS1-Ext LF/oAM-LU217), and for the verification of the 3’ flank double recombination the primer mix oAM-LU218/TS1-Ext RF was utilised. **(C)** Corroboration of double recombination in *ts2* deletion mutants. For the amplification of *hph* the primer combination oAM-LU357/oAM-LU358 was used; for the 5’ flank double recombination was generated by using the primer mixture TS2-Ext LF/oAM-LU217), and for the verification of the 3’ flank double recombination the primer mix oAM-LU218/TS2-Ext RF was utilised.The PCR products were separated on 0.8% agarose gel and the images registered by using a Versadoc system (Bio-Rad).


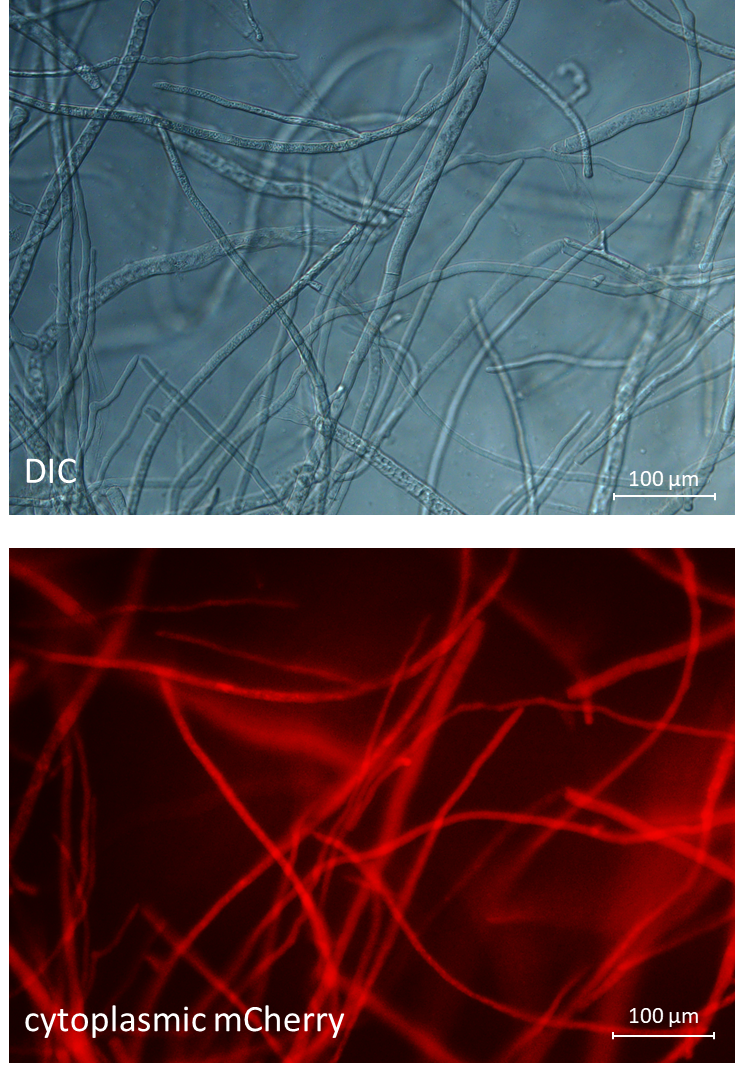


**Supplementary Figure 5.** **Expression of mCherry in *T* *.atroviride* LU132.** The expression of mCherry in mycelia of *T. atroviride*. The mycelia was visualized using an Olympus BX51 compound microscope. Images were captured using an Olympus DP80 digital camera system and processed with the software CellF (Olympus). The experiment was repeated once with similar results using three independent transformants. The upper panel represents the DIC pictures and the lower the mCherry fluorescence using 587nm excitation and 610 emission.


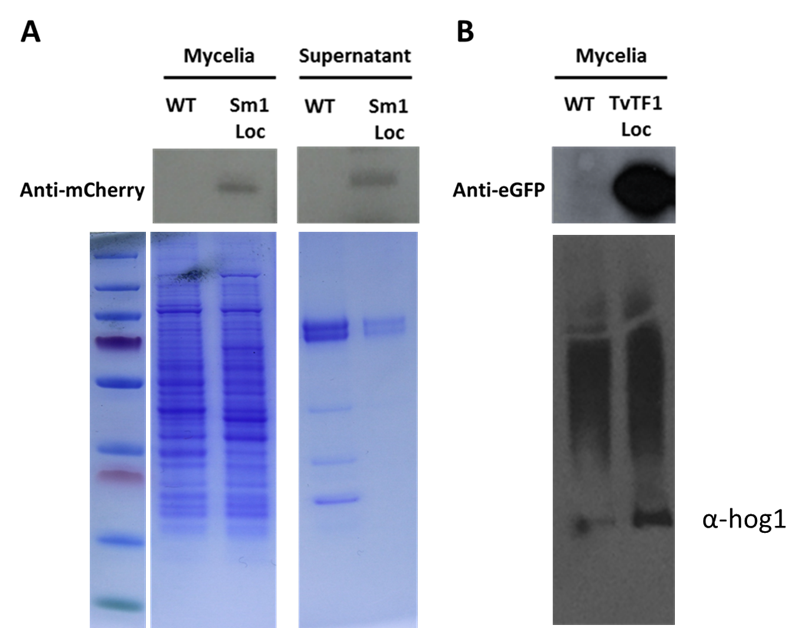


**Supplementary Figure 6.** **Expression of fusions of Sm1-mCherry and TvTF1-eGFP in *T. virens* cells.** Western blot analysis of *T. virens* transformants. Lysates of *T. virens* transformants cells and supernatant (20 µg of total protein) were separated SDS-PAGE, transferred to a nitrocellulose membrane, and probed with an anti-mCherry or anti-eGFP. **(A)** Detection of Sm1-mCherry protein in mycelia and culture supernatant. **(B)** Detection of TvTF1-eGFP protein in mycelia. The loading control in both cases corresponds to parallel gels with identical loading proteins and run at the same time to the western blots shown in the upper part. The control loading for samples in A is a Coomassie staining and for B is a Western blot against anti-Hog1 (y-215) (Santa Cruz Biotechnology, USA).
