# Supplementary material for: TrichoGate: An Improved Vector System for a Large Scale of Functional Analysis of Trichoderma Genes
Source: Front Microbiol. 2019 Dec 10;10:2794. doi: 10.3389/fmicb.2019.02794 (PMC6915037; doi:10.3389/fmicb.2019.02794)
Supplement: Supplementary file 4 [file Table_3.docx]

TrichoGate: An improved Vector System for a Large Scale of Functional Analysis of *Trichoderma*  Genes

Guillermo Nogueria-Lopez^1^, Fabiola Padilla-Arizmendi^1^, Sarah Inwood^1,2^, Sarah Lyne^1^, Johanna M Steyaert ^1,3^, Maria Fernanda Nieto-Jacobo^1,4^, Alison Stewart^1,5^, Artemio Mendoza-Mendoza^1,*^

^1^ Bio-Protection Research Centre, Lincoln University, Lincoln 7647, New Zealand

^2^ Biochemistry Department, University of Otago, Dunedin, New Zealand

^3^ Lincoln Agritech Ltd, PO Box 69133, Lincoln, Christchurch 7460, New Zealand

^4^ Plant & Food Research Gerald St, Lincoln 7608, New Zealand

**^5^**  Foundation For Arable Research (FAR), Templeton, Christchurch 7678, New Zealand

*** Corresponding Author:**Artemio Mendoza-Mendoza
Artemio.mendoza@lincoln.ac.nz

| **Supplementary Table 3A**. TrichoGate strategy for the creation of fluorescent markers | | | | | | | | | | | | | | | | |  |  |  |
| --- | --- | --- | --- | --- | --- | --- | --- | --- | --- | --- | --- | --- | --- | --- | --- | --- | --- | --- | --- |
|  | | |  | | |  | | | | Recognition and cutting *Bsa*I site | | | Target sequence | | | |  |  |  |
| **Promoter** | | |  | | |  | | | |  | | |  | | | |  |  |  |
|  |  |  | Promoter X | | | Left border-BsaI Forward | | | | GGTCTCt **ACAA** | | | Sequence left region primer from promoter X | | | |  |  |  |
|  |  |  | Promoter X | | | Left border-BsaI Reverse | | | | GGTCTCa **CATT** | | | Reverse complementary left region primer from promoter X | | | |  |  |  |
|  |  |  |  | | | | | | | | | | | | | |  |  |  |
| **Marker** | | |  | | | | | | | | | | | | | |  |  |  |
|  |  |  | Marker X | | | Left region-BsaI Forward | | | | GGTCTCt **AATG** | | | Sequence left region primer from marker X | | | |  |  |  |
|  |  |  | Marker X | | | Right region-BsaI Reverse | | | | GGTCTCt **AAGC** | | | Reverse complementary left region primer from marker X | | | |  |  |  |
|  |  |  |  | | | | | | | | | | | | | |  |  |  |
| **Terminator** | | |  | | |  | | | |  | | |  | | | |  |  |  |
|  |  |  | Terminator X | | | Left region-BsaI Forward | | | | GGTCTCt **GCTT** | | | Sequence left region primer from terminator X | | | |  |  |  |
|  |  |  | Terminator X | | | Right region-BsaI Reverse | | | | GGTCTCc **GTTA** | | | Reverse complementary left region primer from terminator X | | | |  |  |  |
|  |  |  |  | | |  | | | |  | | |  | | | |  |  |  |
| **Resistance cassette** | | |  | | | | | | | | | | | | | |  |  |  |
|  |  |  | Resistance cassette X | | | Left region-BsaI Forward | | | | GGTCTCt **TAAC** | | | Sequence left region primer from resistance cassette X | | | |  |  |  |
|  |  |  | Resistance cassette X | | | Right region-BsaI Reverse | | | | GGTCTCa **ACAT** | | | Reverse complementary left region primer from resistance cassette X | | | |  |  |  |
|  |  |  |  | | | | | | | | | | | | | |  |  |  |
| **Example:** | | |  | | | Primer name | | | | Recognition and cutting *Bsa*I site | | | Primer specific sequence (5'--->3')* | | | |  |  |  |
|  | | |  | | |  | | | |  | | |  | | | |  |  |  |
| gpdh promoter | | | Gpdh Fw | | | Left region-BsaI Forward | | | | GGTCTCt **ACAA** | | | AATTCCCGTTCCTGGAAG | | | |  |  |  |
| gpdh promoter | | | Gpdh Rw | | | Right region-BsaI Reverse | | | | GGTCTCc **TTAC** | | | GTAGCTGATTTGT | | | |  |  |  |
| Marker | | | 3xeGFP Fw | | | Left region-BsaI Forward | | | | GGTCTCt **AATG** | | | GTATCAAAGGGCGAAGAGCTG | | | |  |  |  |
| Marker | | | 3xeGFP Rw | | | Right region-BsaI Reverse | | | | GGTCTCt **AAGC** | | | TTATACAACTCGTCCATA | | | |  |  |  |
| Ttrp terminator | | | Ttrp Fw | | | Left region-BsaI Forward | | | | GGTCTCt **GCTT** | | | CTAGTGATTTAATAGCTCC | | | |  |  |  |
| Ttrp terminator | | | Ttrp Rw | | | Right region-BsaI Reverse | | | | GGTCTCc **GTTA** | | | GGTACCTGTGCATTCTGG | | | |  |  |  |
| HygromycinR | | | HygR Fw | | | Left region-BsaI Forward | | | | GGTCTCt **TAAC** | | | CCGTTAACGGAACCCGGTCGG | | | |  |  |  |
| HygromycinR | | | HygR Rw | | | Right region-BsaI Reverse | | | | GGTCTCa **ACAT** | | | TGATATTGAAGGAGCATTTTTG | | | |  |  |  |
|  | | |  | | |  | | | |  | | |  | | | |  |  |  |
| Notes: red Colour lowercase nucleotide; any nucleotide of choice. N; represents any nucleotide depending on the sequence of the target DNA molecule. Colour nucleotides; represents four nucleotide overhangs. | | | | | | | | | | | | | | | | |  |  |  |
| * For the reverse primers use the reverse complementary sequence of the desired gene | | | | | | | | | | | | | | | | |  |  |  |
|  | | |  | | |  | | | |  | | |  | | | |  |  |  |
|  | | |  | | |  | | | |  | | |  | | | |  |  |  |
| \|  \| \| --- \|   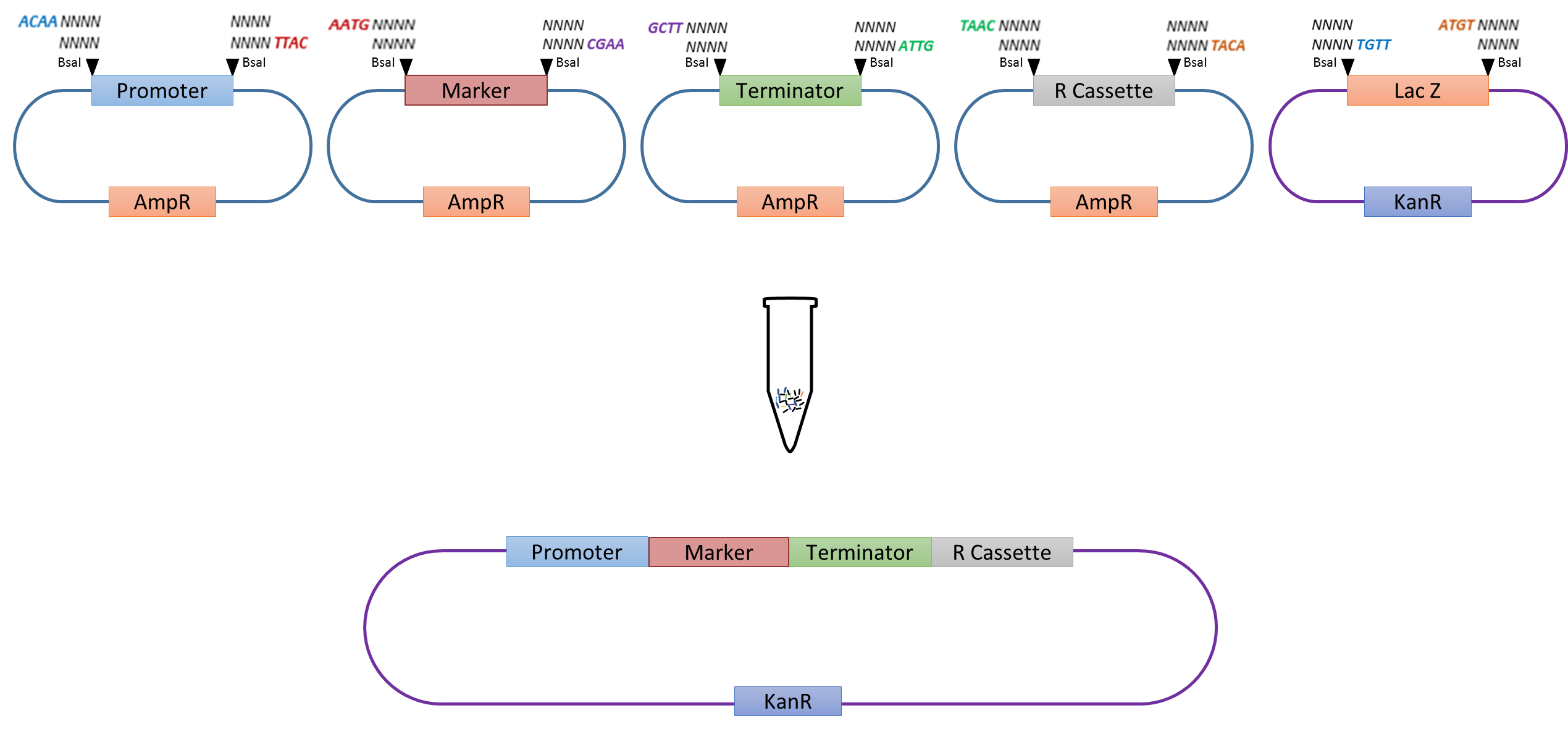 | | |  | | |  | | | |  | | |  | | | |  |  |  |
|  | | |  | | |  | | | |  | | |  | | | |  |  |  |
|  | | |  | | |  | | | |  | | |  | | | |  |  |  |
|  | | |  | | |  | | | |  | | |  | | | |  |  |  |
|  | | |  | | |  | | | |  | | |  | | | |  |  |  |
|  | | |  | | |  | | | |  | | |  | | | |  |  |  |
|  | | |  | | |  | | | |  | | |  | | | |  |  |  |
|  | | |  | | |  | | | |  | | |  | | | |  |  |  |
|  | | |  | | |  | | | |  | | |  | | | |  |  |  |
|  | | |  | | |  | | | |  | | |  | | | |  |  |  |
|  | | |  | | |  | | | |  | | |  | | | |  |  |  |
|  | | |  | | |  | | | |  | | |  | | | |  |  |  |
|  | | |  | | |  | | | |  | | |  | | | |  |  |  |
|  | | |  | | |  | | | |  | | |  | | | |  |  |  |
|  | | |  | | |  | | | |  | | |  | | | |  |  |  |
|  | | |  | | |  | | | |  | | |  | | | |  |  |  |
|  | | |  | | |  | | | |  | | |  | | | |  |  |  |
|  | | |  | | |  | | | |  | | |  | | | |  |  |  |
|  | | |  | | |  | | | |  | | |  | | | |  |  |  |
|  | | |  | | |  | | | |  | | |  | | | |  |  |  |
|  | |  | | | | | |  |  | | | | | | |  |  |  |  |
|  | |  | | | | | |  |  | | | | | | |  |  |  |  |
| **Supplementary Table 3B**. TrichoGate strategy for gene overexpression | | | | | | | | | | | | | |  | | | |  |  |
|  |  | | | |  | | | | | | Recognition and cutting *Bsa*I site | | | Target sequence | | | |  |  |
| **Promoter** |  | | | |  | | | | | |  | | |  | | | |  |  |
|  | Promoter X | | | | Left border-BsaI Forward | | | | | | GGTCTCt **ACAA** | | | Sequence left region primer from promoter X | | | |  |  |
|  | Promoter X | | | | Left border-BsaI Reverse | | | | | | GGTCTCa **CATT** | | | Reverse complementary left region primer from promoter X | | | |  |  |
|  |  | | | |  | | | | | |  | | |  | | | |  |  |
| **Marker** |  | | | |  | | | | | |  | | |  | | | |  |  |
|  | Gene ORF X | | | | Left region-BsaI Forward | | | | | | GGTCTCt **AATG** | | | Sequence left region primer from gene X | | | |  |  |
|  | Gene ORF X | | | | Right region-BsaI Reverse | | | | | | GGTCTCt **AAGC** | | | Reverse complementary left region primer from gene X | | | |  |  |
|  |  | | | |  | | | | | |  | | |  | | | |  |  |
| **Terminator** |  | | | |  | | | | | |  | | |  | | | |  |  |
|  | Terminator X | | | | Left region-BsaI Forward | | | | | | GGTCTCt **GCTT** | | | Sequence left region primer from terminator X | | | |  |  |
|  | Terminator X | | | | Right region-BsaI Reverse | | | | | | GGTCTCc **GTTA** | | | Reverse complementary left region primer from terminator X | | | |  |  |
|  |  | | | |  | | | | | |  | | |  | | | |  |  |
| **Resistance cassette** |  | | | |  | | | | | |  | | |  | | | |  |  |
|  | Resistance cassette X | | | | Left region-BsaI Forward | | | | | | GGTCTCt **TAAC** | | | Sequence left region primer from resistance cassette X | | | |  |  |
|  | Resistance cassette X | | | | Right region-BsaI Reverse | | | | | | GGTCTCa **ACAT** | | | Reverse complementary left region primer from resistance cassette X | | | |  |  |
|  |  | | | |  | | | | | |  | | |  | | | |  |  |
| **Example:** | Primer name | | | |  | | | | | | Recognition and cutting *Bsa*I site | | | Primer specific sequence (5'--->3')* | | | |  |  |
|  |  | | | |  | | | | | |  | | |  | | | |  |  |
| gpdh promoter | Gpdh Fw | | | | Left region-BsaI Forward | | | | | | GGTCTCt **ACAA** | | | AATTCCCGTTCCTGGAAG | | | |  |  |
| gpdh promoter | Gpdh Rw | | | | Right region-BsaI Reverse | | | | | | GGTCTCc **TTAC** | | | GTAGCTGATTTGT | | | |  |  |
| ORF | Fw | | | | Left region-BsaI Forward | | | | | | GGTCTCt **AATG** | | | NNNNNNNNNNNNNN | | | |  |  |
| ORF | Rw | | | | Right region-BsaI Reverse | | | | | | GGTCTCt **AAGC** | | | NNNNNNNNNNNNNN | | | |  |  |
| Ttrp terminator | Ttrp Fw | | | | Left region-BsaI Forward | | | | | | GGTCTCt **GCTT** | | | CTAGTGATTTAATAGCTCC | | | |  |  |
| Ttrp terminator | Ttrp Rw | | | | Right region-BsaI Reverse | | | | | | GGTCTCc **GTTA** | | | GGTACCTGTGCATTCTGG | | | |  |  |
| HygromycinR | HygR Fw | | | | Left region-BsaI Forward | | | | | | GGTCTCt **TAAC** | | | CCGTTAACGGAACCCGGTCGG | | | |  |  |
| HygromycinR | HygR Rw | | | | Right region-BsaI Reverse | | | | | | GGTCTCa **ACAT** | | | TGATATTGAAGGAGCATTTTTG | | | |  |  |
|  |  | | | |  | | | | | |  | | |  | | | |  |  |
| Notes: red Colour lowercase nucleotide; any nucleotide of choice. N; represents any nucleotide depending on the sequence of the target DNA molecule. Colour nucleotides; represents four nucleotide overhangs. | | | | | | | | | | | | | | | | | |  |  |
| * For the reverse primers use the reverse complementary sequence of the desired gene | | | | | | | | | | | | | | | | | |  |  |
|  |  | | | |  | | | | | |  | | |  | | | |  |  |
| 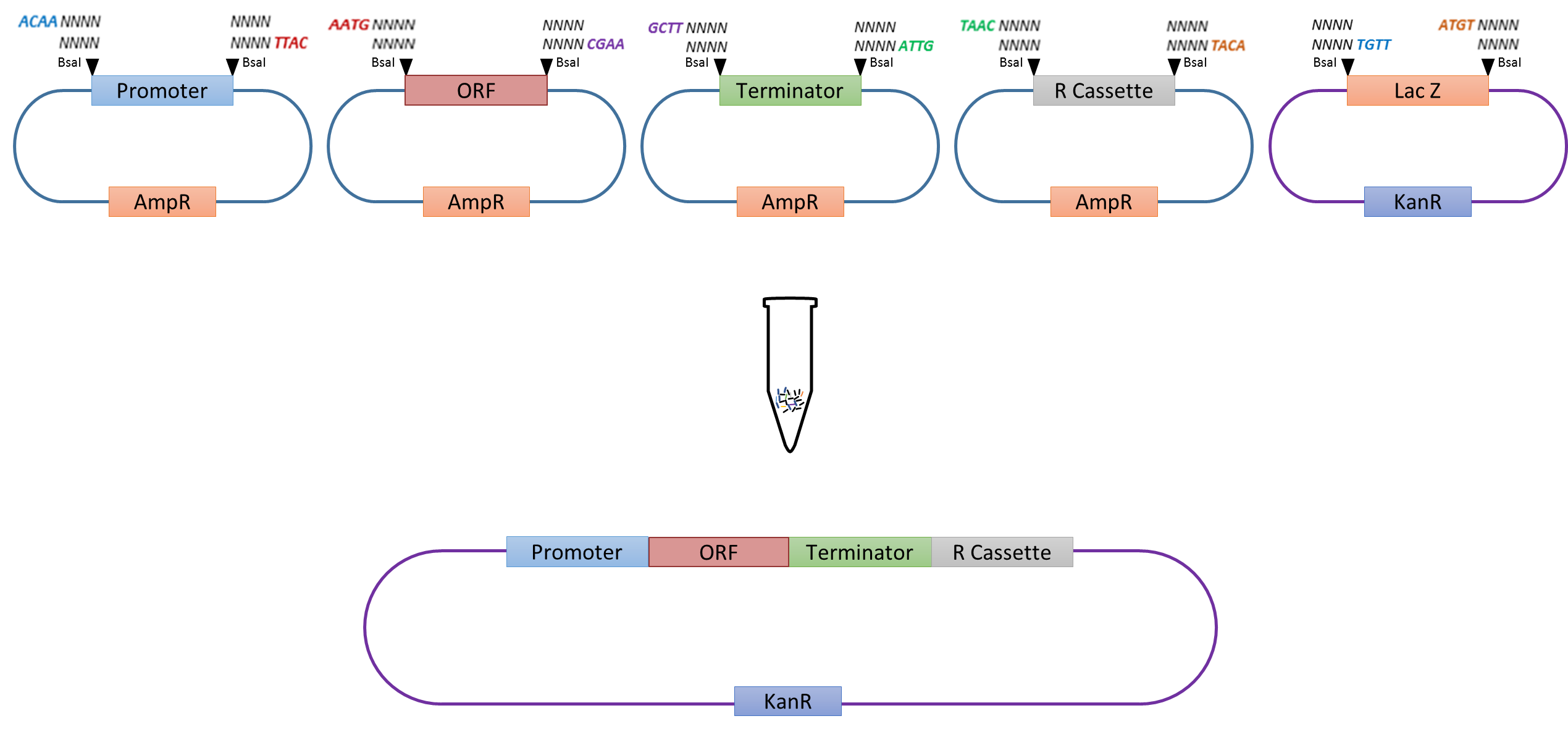   \|  \| \| --- \| |  | | | |  | | | | | |  | | |  | | | |  |  |
|  |  | | | |  | | | | | |  | | |  | | | |  |  |
|  |  | | | |  | | | | | |  | | |  | | | |  |  |
|  |  | | | |  | | | | | |  | | |  | | | |  |  |
|  |  | | | |  | | | | | |  | | |  | | | |  |  |
|  |  | | | |  | | | | | |  | | |  | | | |  |  |
|  |  | | | |  | | | | | |  | | |  | | | |  |  |
|  |  | | | |  | | | | | |  | | |  | | | |  |  |
|  |  | | | |  | | | | | |  | | |  | | | |  |  |
|  |  | | | |  | | | | | |  | | |  | | | |  |  |
|  |  | | | |  | | | | | |  | | |  | | | |  |  |
|  |  | | | |  | | | | | |  | | |  | | | |  |  |
|  |  | | | |  | | | | | |  | | |  | | | |  |  |
|  |  | | | |  | | | | | |  | | |  | | | |  |  |
|  |  | | | |  | | | | | |  | | |  | | | |  |  |
|  |  | | | |  | | | | | |  | | |  | | | |  |  |
|  |  | | | |  | | | | | |  | | |  | | | |  |  |
|  |  | | | |  | | | | | |  | | |  | | | |  |  |
|  |  | | | |  | | | | | |  | | |  | | | |  |  |
|  |  | | | |  | | | | | |  | | |  | | | |  |  |
|  |  | | | |  | | | | | |  | | |  | | | |  |  |
|  |  | | | |  | | | | | |  | | |  | | | |  |  |
|  |  | | | |  | | | | | |  | | |  | | | |  |  |
|  |  | | | |  | | | | | |  | | |  | | | |  |  |
| **Supplementary Table 3C**. TrichoGate strategy for gene deletion constructs | | | | | | | | | | | | | | | | | | |  |
|  | | | |  | | |  | | | | | Recognition and cutting *Bsa*I site | | | Target sequence | | | | |
| **Gene flanks** | | | |  | | |  | | | | |  | | |  | | | | |
|  |  |  |  | Left flank gene X | | | Left flank-BsaI Forward | | | | | GGTCTCt **ACAA** | | | Sequence left flank primer from gene X | | | | |
|  |  |  |  | Left flank gene X | | | Left flank-BsaI Reverse | | | | | GGTCTCa **CATT** | | | Reverse complementary left flank primer from gene X | | | | |
|  |  |  |  | Right flank gene X | | | Right flank-BsaI Forward | | | | | GGTCTCt **GCTT** | | | Sequence right borde primer from gene X | | | | |
|  |  |  |  | Right flank gene X | | | Right flank-BsaI Reverse | | | | | GGTCTCa **ACAT** | | | Reverse complementary right flank primer from gene X | | | | |
|  |  |  |  |  | | |  | | | | |  | | |  | | | | |
| **Resistance cassette** | | | |  | | |  | | | | |  | | |  | | | | |
|  |  |  |  | Resistance cassette X | | | Left region-BsaI Forward | | | | | GGTCTCt **AATG** | | | Sequence left region resistance cassette | | | | |
|  |  |  |  | Resistance cassette X | | | Right region-BsaI Reverse | | | | | GGTCTCt **AAGC** | | | Reverse complementary left region primer from resistance cassette | | | | |
|  |  |  |  |  | | |  | | | | |  | | |  | | | | |
| **Example:** | | | | Primer name | | |  | | | | | Recognition and cutting *Bsa*I site | | | Primer specific sequence (5'--->3')* | | | | |
|  | | | |  | | |  | | | | |  | | |  | | | | |
| Left flank gene X | | | | LF Fw | | | Left flank-BsaI Forward | | | | | GGTCTCt **ACAA** | | | NNNNNNNNNNNNNNNN | | | | |
| Left flank gene X | | | | LF Rw | | | Left flank-BsaI Reverse | | | | | GGTCTCa **CATT** | | | NNNNNNNNNNNNNNNN | | | | |
| Right flank gene X | | | | RF Fw | | | Right flank-BsaI- Forward | | | | | GGTCTCt **GCTT** | | | NNNNNNNNNNNNNNNN | | | | |
| Right flank gene X | | | | RF Rw | | | Right flank-BsaI Reverse | | | | | GGTCTCa **ACAT** | | | NNNNNNNNNNNNNNNN | | | | |
| HygromycinR deletion | | | | HygR ∆ Fw | | | Left region-BsaI Forward | | | | | GGTCTCt **AATG** | | | TGATATTGAAGGAGCATTTTTG | | | | |
| HygromycinR deletion | | | | HygR ∆ Rw | | | Right region-BsaI Reverse | | | | | GGTCTCt **AAGC** | | | CCGTTAACGGAACCCGGTCGG | | | | |
|  | | | |  | | |  | | | | |  | | |  | | | | |
| Notes: red Colour lowercase nucleotide; any nucleotide of choice. N; represents any nucleotide depending on the sequence of the target DNA molecule. Colour nucleotides; represents four nucleotide overhangs. | | | | | | | | | | | | | | | | | | |  |
| * For the revese primers use the reverse complementary sequence of the desired gene | | | | | | | | | | | | | | | | | | |  |
|  | | | |  | | |  | | | | |  | | |  | | | | |
|  | | | |  | | |  | | | | |  | | |  | | | | |
| \|  \| \| --- \| | | | |  | | |  | | | | |  | | |  | | | | |
|  | | | |  | | |  | | | | |  | | |  | | | | |
|  | | | |  | | |  | | | | |  | | |  | | | | |
|  | | | |  | | |  | | | | |  | | |  | | | | |
|  | | | |  | | |  | | | | |  | | |  | | | | |
|  | | | |  | | |  | | | | |  | | |  | | | | |
|  | | | |  | | |  | | | | |  | | |  | | | | |
|  | | | |  | | |  | | | | |  | | |  | | | | |
|  | | | |  | | |  | | | | |  | | |  | | | | |
|  | | | |  | | |  | | | | |  | | | 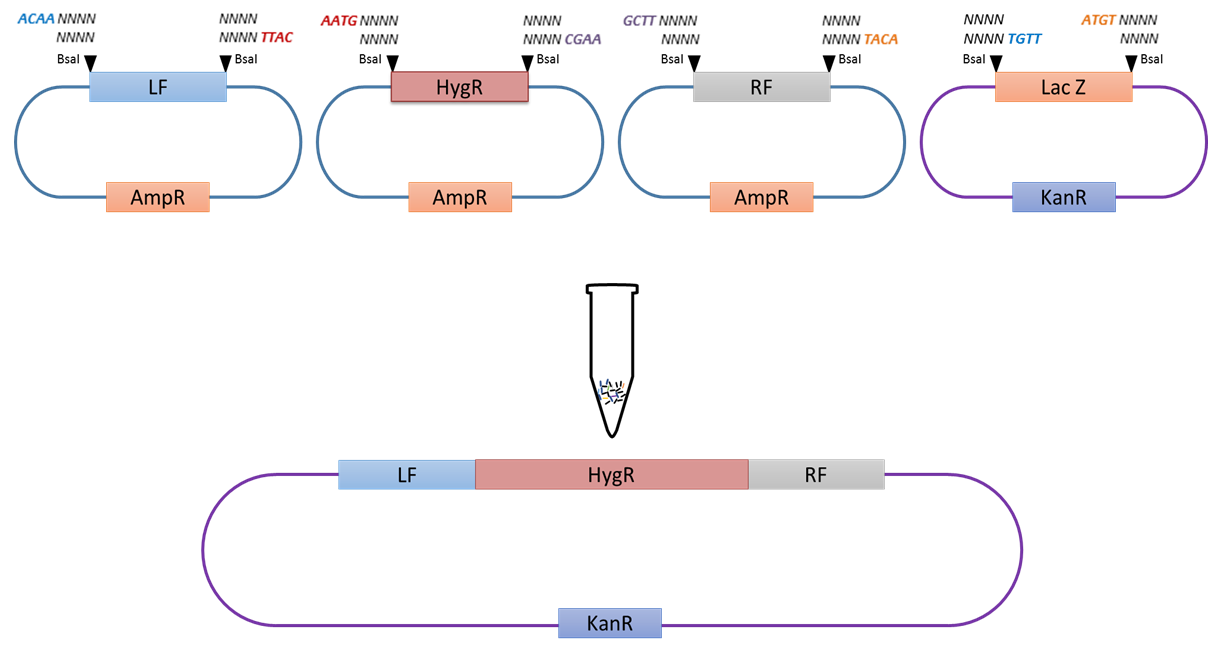 | | | | |
|  | | | |  | | |  | | | | |  | | |  | | | | |
|  | | | |  | | |  | | | | |  | | |  | | | | |
|  | | | |  | | |  | | | | |  | | |  | | | | |
|  | | | |  | | |  | | | | |  | | |  | | | | |
|  | | | |  | | |  | | | | |  | | |  | | | | |
|  | | | |  | | |  | | | | |  | | |  | | | | |
|  | | | |  | | |  | | | | |  | | |  | | | | |
|  | | | |  | | |  | | | | |  | | |  | | | | |
|  | | | |  | | |  | | | | |  | | |  | | | | |
|  | | | |  | | |  | | | | |  | | |  | | | | |
|  | | | |  | | |  | | | | |  | | |  | | | | |
|  | | | |  | | |  | | | | |  | | |  | | | | |
|  | | | |  | | |  | | | | |  | | |  | | | | |
|  | | | |  | | |  | | | | |  | | |  | | | | |
|  | | | |  | | |  | | | | |  | | |  | | | | |
|  | | | |  | | |  | | | | |  | | |  | | | | |
|  | | | |  | | |  | | | | |  | | |  | | | | |
|  | | | |  | | |  | | | | |  | | |  | | | | |
|  | | | |  | | |  | | | | |  | | |  | | | | |
|  | | | |  | | |  | | | | |  | | |  | | | | |

| **Supplementary Table 3D**. TrichoGate strategy for gene complementation constructs | | | | | | | | | | | | | | | | | | |  |
| --- | --- | --- | --- | --- | --- | --- | --- | --- | --- | --- | --- | --- | --- | --- | --- | --- | --- | --- | --- |
|  |  | | | | | |  | | | | Recognition and cutting *Bsa*I site | | | | Target sequence | | | |  |
| **Gene** |  | | | | | |  | | | |  | | | |  | | | |  |
|  | Gene ORF X | | | | | | Left border-BsaI Forward | | | | GGTCTCt **ACAA** | | | | Sequence left region primer from gene X | | | |  |
|  | Gene ORF X | | | | | | Left border-BsaI Reverse | | | | GGTCTCa **GTTA** | | | | Reverse complementary left region primer from gene X | | | |  |
|  |  | | | | | |  | | | |  | | | |  | | | |  |
| **Resistance cassette** | **Resistance cassette** | | | | | |  | | | |  | | | |  | | | |  |
|  | Resistance cassette X | | | | | | Left region-BsaI Forward | | | | GGTCTCt **TAAC** | | | | Sequence left region resistance cassette | | | |  |
|  | Resistance cassette X | | | | | | Right region-BsaI Reverse | | | | GGTCTCa **ACAT** | | | | Reverse complementary left region primer from resistance cassette | | | |  |
|  |  | | | | | |  | | | |  | | | |  | | | |  |
| **Example:** | Primer name | | | | | |  | | | | Recognition and cutting *Bsa*I site | | | | Primer specific sequence (5'--->3')* | | | |  |
|  |  | | | | | |  | | | |  | | | |  | | | |  |
| Comp gene X | Fw Comp | | | | | | Left region-BsaI Forward | | | | GGTCTCt **ACAA** | | | | NNNNNNNNNNNNNNNNNN | | | |  |
| Comp gene X | Rw Comp | | | | | | Left region-BsaI Reverse | | | | GGTCTCc **GTTA** | | | | NNNNNNNNNNNNNNNNNN | | | |  |
| CarboxinR | CbxR Fw | | | | | | Left region-BsaI Forward | | | | GGTCTCt **TAAC** | | | | CGTCGTAACGAACAAGATCTCTC | | | |  |
| CarboxinR | CbxR Rw | | | | | | Right region-BsaI Reverse | | | | GGTCTCa **ACAT** | | | | GTGTCTGTATGTTAAAAACTGC | | | |  |
|  |  | | | | | |  | | | |  | | | |  | | | |  |
| Notes: red Colour lowercase nucleotide; any nucleotide of choice. N; represents any nucleotide depending on the sequence of the target DNA molecule. Colour nucleotides; represents four nucleotide overhangs. | | | | | | | | | | | | | | | | | | |  |
| * For the reverse primers use the reverse complementary sequence of the desired gene | | | | | | | | | | | | | | | | | | |  |
|  |  | | | | | |  | | | |  | | | |  | | | |  |
|  |  | | | | | |  | | | |  | | | |  | | | |  |
| \|  \| \| --- \| |  | | | | | |  | | | |  | | | |  | | | |  |
|  |  | | | | | |  | | | |  | | | |  | | | |  |
|  |  | | | | | |  | | | |  | | | |  | | | |  |
|  |  | | | | | |  | | | |  | | | |  | | | |  |
|  |  | | | | | |  | | | |  | | | |  | | | |  |
|  |  | | | | | | 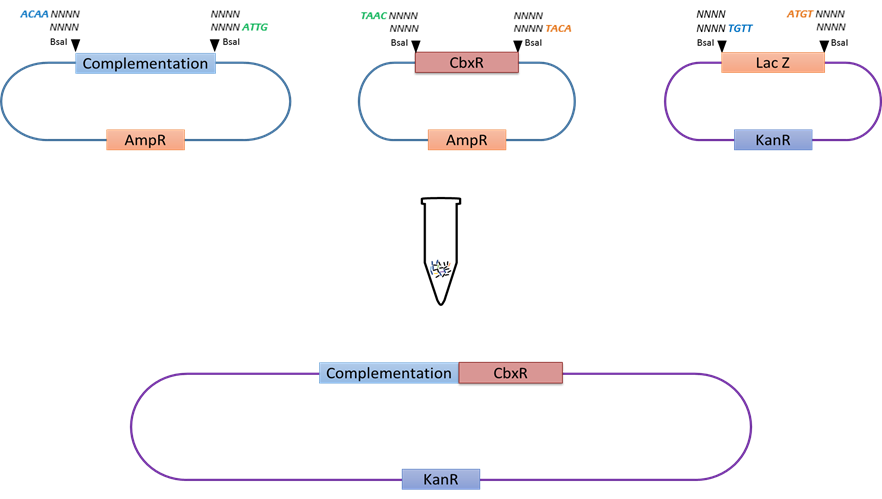 | | | |  | | | |  | | | |  |
|  |  | | | | | |  | | | |  | | | |  | | | |  |
|  |  | | | | | |  | | | |  | | | |  | | | |  |
|  |  | | | | | |  | | | |  | | | |  | | | |  |
|  |  | | | | | |  | | | |  | | | |  | | | |  |
|  |  | | | | | |  | | | |  | | | |  | | | |  |
|  |  | | | | | |  | | | |  | | | |  | | | |  |
|  |  | | | | | |  | | | |  | | | |  | | | |  |
|  |  | | | | | |  | | | |  | | | |  | | | |  |
|  |  | | | | | |  | | | |  | | | |  | | | |  |
|  |  | | | | | |  | | | |  | | | |  | | | |  |
|  |  | | | | | |  | | | |  | | | |  | | | |  |
|  |  | | | | | |  | | | |  | | | |  | | | |  |
|  |  | | | | | |  | | | |  | | | |  | | | |  |
|  |  | | | | | |  | | | |  | | | |  | | | |  |
|  |  | | | | | |  | | | |  | | | |  | | | |  |
|  |  | | | | | |  | | | |  | | | |  | | | |  |
|  |  | | | | | |  | | | |  | | | |  | | | |  |
|  |  | | | | | |  | | | |  | | | |  | | | |  |
|  |  | | | | | |  | | | |  | | | |  | | | |  |
|  |  | | | | | |  | | | |  | | | |  | | | |  |
|  |  | | | | | |  | | | |  | | | |  | | | |  |
|  |  | | | | | |  | | | |  | | | |  | | | |  |
|  |  | | | | | |  | | | |  | | | |  | | | |  |
|  |  | | | | | |  | | | |  | | | |  | | | |  |
| **Supplementary Table 3E**. TrichoGate strategy for C-terminal fusion with fluorescent markers containing a linker | | | | | | | | | | | | | | | | | |  |  |
|  | | |  | |  | | | | Recognition and cutting *Bsa*I site | | | Target sequence | | | | |  |  |  |
| **Promoter** | | |  | |  | | | |  | | |  | | | | |  |  |  |
|  |  |  | Promoter X | | Left border-BsaI Forward | | | | GGTCTCt **ACAA** | | | Sequence left region primer from promoter X | | | | |  |  |  |
|  |  |  | Promoter X | | Left border-BsaI Reverse | | | | GGTCTCa **CATT** | | | Reverse complementary left region primer from promoter X | | | | |  |  |  |
|  |  |  |  | |  | | | |  | | |  | | | | |  |  |  |
| **Gene** | | |  | |  | | | |  | | |  | | | | |  |  |  |
|  |  |  | Gene ORF X | | Left region-BsaI Forward | | | | GGTCTCt **AATG** | | | N-terminal region primer from gene X | | | | |  |  |  |
|  |  |  | Gene ORF X | | Right region-BsaI Reverse | | | | GGTCTCc **CGGC** | | | C-terminal region primer from gene X | | | | |  |  |  |
|  |  |  |  | |  | | | |  | | |  | | | | |  |  |  |
|  |  |  | **Note: Start codon is included in the overhang sequence, thus needs to be ignoredfrom gene X . For the fusion in C-terminal region the stop codon from gene X NEEDS TO BE ignored** | | | | | | | | | | | | | | |  |  |
|  |  |  |  | |  | | | |  | | |  | | | | |  |  |  |
| **Linker-Marker** | | |  | |  | | | |  | | |  | | | | |  |  |  |
|  |  |  | Marker X | | Left region-BsaI Forward | | | | GGTCTCa **GCCG** | | | Linker + N-terminal region primer from marker X | | | | |  |  |  |
|  |  |  | Marker X | | Right region-BsaI Reverse | | | | GGTCTCt **AAGC** | | | C-terminal region primer from marker X | | | | |  |  |  |
|  |  |  |  | |  | | | |  | | |  | | | | |  |  |  |
| **Terminator** | | |  | |  | | | |  | | |  | | | | |  |  |  |
|  |  |  | Terminator X | | Left region-BsaI Forward | | | | GGTCTCt **GCTT** | | | Sequence left region primer from terminator X | | | | |  |  |  |
|  |  |  | Terminator X | | Right region-BsaI Reverse | | | | GGTCTCc **GTTA** | | | Reverse complementary left region primer from terminator X | | | | |  |  |  |
|  |  |  |  | |  | | | |  | | |  | | | | |  |  |  |
| **Resistance cassette** | | |  | |  | | | |  | | |  | | | | |  |  |  |
|  |  |  | Resistance cassette X | | Left region-BsaI Forward | | | | GGTCTCt **TAAC** | | | Sequence left region primer from resistance cassette X | | | | |  |  |  |
|  |  |  | Resistance cassette X | | Right region-BsaI Reverse | | | | GGTCTCa **ACAT** | | | Reverse complementary left region primer from resistance cassette X | | | | |  |  |  |
|  |  |  |  | |  | | | |  | | |  | | | | |  |  |  |
| **Example:** | | | Primer name | |  | | | | Recognition and cutting *Bsa*I site | | | Primer specific sequence (5'--->3') | | | | |  |  |  |
|  | | |  | |  | | | |  | | |  | | | | |  |  |  |
| Tef1 promoter | | | Tef1 Fw | | Left region-BsaI Forward | | | | GGTCTCt **ACAA** | | | TACCAACACAGAGGGACGCGC | | | | |  |  |  |
| Tef1 promoter | | | Tef1 Rw | | Right region-BsaI Reverse | | | | GGTCTCa **CATT** | | | TTTTGCGGTTTGTGAAATG | | | | |  |  |  |
| Sm1 | | | Sm1 ORF Fw | | Left region-BsaI Forward | | | | GGTCTCt **AATG** | | | CAACTGTCCAACATCTTCACTCTC | | | | |  |  |  |
| Sm1 | | | Sm1 ORF Rw | | Right region-BsaI Reverse | | | | GGTCTCc **CGGC** | | | GAGTCCGCAGTTCTTAACAGGAA * | | | | |  |  |  |
| Linker-mCherry | | | mCherry Fw | | Linker + left region-BsaI Reverse | | | | GGTCTCa **GCCG** | | | **GATCTGCTGGTTCTGCTGCTGGTTCTGGTGAATTC** ATGGTCAGCAAGGGCGAAGAA | | | | |  |  |  |
| Linker-mCherry | | | mCherry Rw | | Right region-BsaI Reverse | | | | GGTCTCt **AAGC** | | | TTACTTGTAGAGTTCGTC | | | | |  |  |  |
| T-nos terminator | | | T-nos Fw | | Left region-BsaI Forward | | | | GGTCTCt **GCTT** | | | GGCGCGCCGGCCGCCCGGC | | | | |  |  |  |
| T-nos terminator | | | T-nos Rw | | Right region-BsaI Reverse | | | | GGTCTCc **GTTA** | | | AATTCTCATGTTTGACAGC | | | | |  |  |  |
| HygromycinR | | | HygR Fw | | Left region-BsaI Forward | | | | GGTCTCt **TAAC** | | | CCGTTAACGGAACCCGGTCGG | | | | |  |  |  |
| HygromycinR | | | HygR Rw | | Right region-BsaI Reverse | | | | GGTCTCa **ACAT** | | | TGATATTGAAGGAGCATTTTTG | | | | |  |  |  |
|  | | |  | |  | | | |  | | |  | | | | |  |  |  |
| Notes: red Colour lowercase nucleotide; any nucleotide of choice. N; represents any nucleotide depending on the sequence of the target DNA molecule. Yellow nucleotide; represents linker sequence. Colour nucleotides except yellow; represents four nucleotide overhangs. | | | | | | | | | | | | | | | | | |  |  |
| * For the reverseprimers use the reverse complementary sequence of the desired gene | | | | | | | | | | | | | | | | | |  |  |
|  | | |  | |  | | | |  | | |  | | | | |  |  |  |
|  | | |  | |  | | | |  | | |  | | | | |  |  |  |
| \|  \| \| --- \| | | |  | |  | | | |  | | |  | | | | |  |  |  |
|  | | |  | |  | | | |  | | |  | | | | |  |  |  |
|  | | |  | |  | | | |  | | |  | | | | |  |  |  |
|  | | |  | |  | | | |  | | |  | | | | |  |  |  |
|  | | |  | |  | | | |  | | |  | | | | |  |  |  |
|  | | |  | |  | | | |  | | |  | | | | |  |  |  |
|  | | |  | |  | | | |  | | |  | | | | |  |  |  |
|  | | |  | |  | | | |  | | |  | | | | |  |  |  |
|  | | |  | |  | | | |  | | |  | | | | |  |  |  |
|  | | |  | |  | | | |  | | |  | | | | |  |  |  |
|  | | |  | |  | | | |  | | |  | | | | |  |  |  |
|  | | |  | |  | | | |  | | |  | | | | |  |  |  |
|  | | |  | |  | | | |  | | |  | | | | |  |  |  |
|  | | |  | |  | | | |  | | |  | | | | |  |  |  |
|  | | |  | |  | | | |  | | |  | | | | |  |  |  |
|  | | |  | |  | | | |  | | |  | | | | |  |  |  |
|  | | |  | |  | | | |  | | |  | | | | |  |  |  |
|  | | |  | |  | | | |  | | |  | | | | |  |  |  |
|  | | |  | |  | | | |  | | |  | | | | |  |  |  |
|  | | |  | |  | | | |  | | |  | | | | |  |  |  |
|  | | |  | |  | | | |  | | | 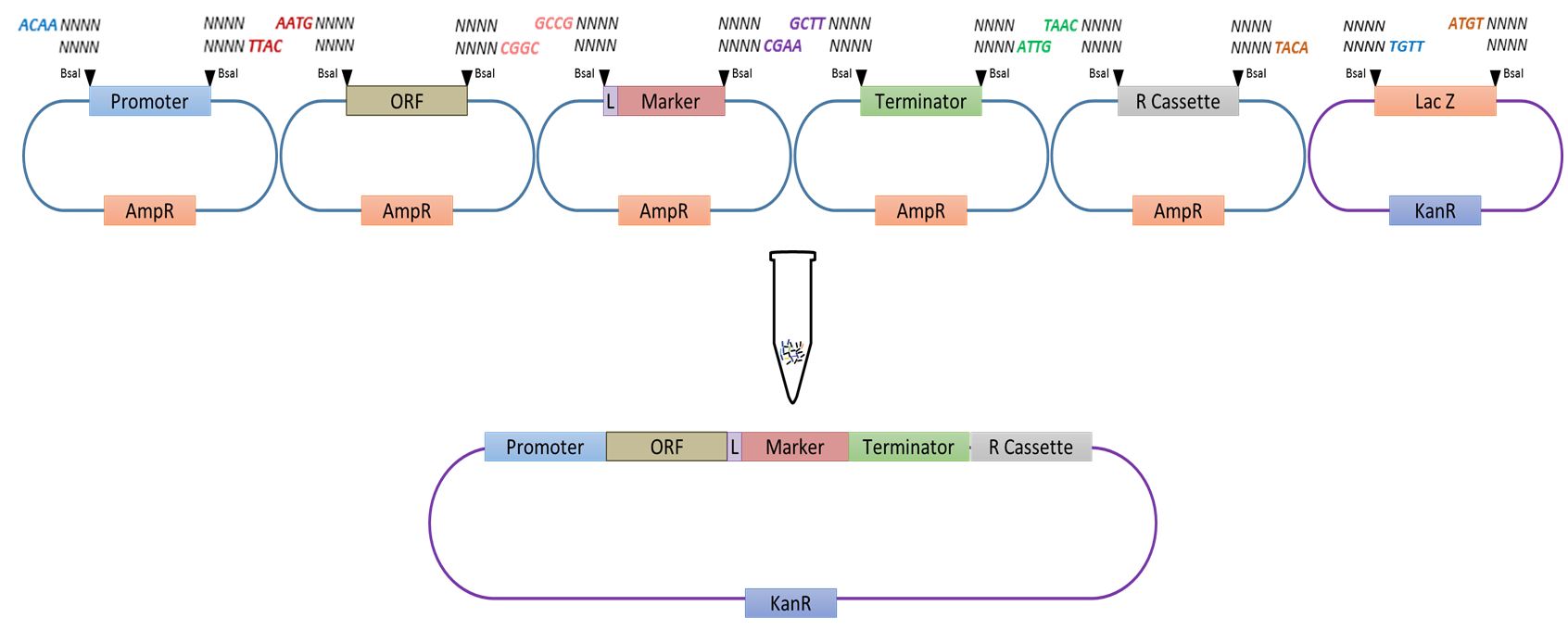 | | | | |  |  |  |
|  | | |  | |  | | | |  | | |  | | | | |  |  |  |
|  | | |  | |  | | | |  | | |  | | | | |  |  |  |
| **Supplementary Table 3F**. TrichoGate strategy for N-terminal fusion with fluorescent markers containing a linker | | | | | | | | | | | | | | | | | | |  |
|  | |  | | | |  | | | | Recognition and cutting *Bsa*I site | | | Target sequence | | | | | |  |
| **Promoter** | |  | | | |  | | | |  | | |  | | | | | |  |
|  |  | Promoter X | | | | Left border-BsaI Forward | | | | GGTCTCt **ACAA** | | | Sequence left region primer from promoter X | | | | | |  |
|  |  | Promoter X | | | | Left border-BsaI Reverse | | | | GGTCTCa **CATT** | | | Reverse complementary left region primer from promoter X | | | | | |  |
|  |  |  | | | |  | | | |  | | |  | | | | | |  |
| **Linker-Marker** | |  | | | |  | | | |  | | |  | | | | | |  |
|  |  | Marker X | | | | Left region-BsaI Forward | | | | GGTCTCt **AATG** | | | N-terminal region primer from marker X | | | | | |  |
|  |  | Marker X | | | | Right region-BsaI Reverse | | | | GGTCTCa **TAGC** | | | Linker + C-terminal region primer from marker X | | | | | |  |
|  |  |  | | | |  | | | |  | | |  | | | | | |  |
| **Gene** | |  | | | |  | | | |  | | |  | | | | | |  |
|  |  | Gene ORF X | | | | Left region-BsaI Forward | | | | GGTCTCt **GCTA** | | | Sequence left region primer from gene X | | | | | |  |
|  |  | Gene ORF X | | | | Right region-BsaI Reverse | | | | GGTCTCt **AAGC** | | | Reverse complementary left region primer from gene X | | | | | |  |
|  |  |  | | | |  | | | |  | | |  | | | | | |  |
| **Terminator** | |  | | | |  | | | |  | | |  | | | | | |  |
|  |  | Terminator X | | | | Left region-BsaI Forward | | | | GGTCTCt **GCTT** | | | Sequence left region primer from terminator X | | | | | |  |
|  |  | Terminator X | | | | Right region-BsaI Reverse | | | | GGTCTCc **GTTA** | | | Reverse complementary left region primer from terminator X | | | | | |  |
|  |  |  | | | |  | | | |  | | |  | | | | | |  |
| **Resistance cassette** | |  | | | |  | | | |  | | |  | | | | | |  |
|  |  | Resistance cassette X | | | | Left region-BsaI Forward | | | | GGTCTCt **TAAC** | | | Sequence left region primer from resistance cassette X | | | | | |  |
|  |  | Resistance cassette X | | | | Right region-BsaI Reverse | | | | GGTCTCa **ACAT** | | | Reverse complementary left region primer from resistance cassette X | | | | | |  |
|  |  |  | | | |  | | | |  | | |  | | | | | |  |
| **Example:** | | Primer name | | | |  | | | | Recognition and cutting *Bsa*I site | | | Primer specific sequence (5'--->3') | | | | | |  |
| Tef1 promoter | | Tef1 Fw | | | | Left region-BsaI Forward | | | | GGTCTCt **ACAA** | | | TACCAACACAGAGGGACGCGC | | | | | |  |
| Tef1 promoter | | Tef1 Rw | | | | Right region-BsaI Reverse | | | | GGTCTCa **CATT** | | | TTTTGCGGTTTGTGAAATG | | | | | |  |
| eGFP-Linker | | eGFP Fw | | | | Left region-BsaI Reverse + Linker | | | | GGTCTCt **AATG** | | | GTCAGCAAGGGCGAA | | | | | |  |
| eGFP-Linker | | eGFP Rw | | | | Right region-BsaI Reverse | | | | GGTCTCa **TAGC** | | | **GAATTCACCAGAACCAGCAGCAGAACCAGCAGAACC** CTTATACAACTCGTCCATGCC | | | | | |  |
| Gene ORF X | | ORF Fw | | | | Left region-BsaI Forward | | | | GGTCTCt **GCTA** | | | NNNNNNNNNNNNNNNNNN | | | | | |  |
| Gene ORF X | | ORF Rw | | | | Right region-BsaI Reverse | | | | GGTCTCt **AAGC** | | | NNNNNNNNNNNNNNNNNN | | | | | |  |
| T-nos terminator | | T-nos Fw | | | | Left region-BsaI Forward | | | | GGTCTCt **GCTT** | | | GGCGCGCCGGCCGCCCGGC | | | | | |  |
| T-nos terminator | | T-nos Rw | | | | Right region-BsaI Reverse | | | | GGTCTCc **GTTA** | | | AATTCTCATGTTTGACAGC | | | | | |  |
| HygromycinR | | HygR Fw | | | | Left region-BsaI Forward | | | | GGTCTCt **TAAC** | | | CCGTTAACGGAACCCGGTCGG | | | | | |  |
| HygromycinR | | HygR Rw | | | | Right region-BsaI Reverse | | | | GGTCTCa **ACAT** | | | TGATATTGAAGGAGCATTTTTG | | | | | |  |
| Notes: red Colour lowercase nucleotide; any nucleotide of choice. N; represents any nucleotide depending on the sequence of the target DNA molecule. Yellow nucleonite; represents linker sequence. Colour nucleotides except yellow; represents four nucleotide overhangs. | | | | | | | | | | | | | | | | | | |  |
| * For the revese primers use the reverse complementary sequence of the desired gene | | | | | | | | | | | | | | | | | | |  |
|  | |  | | | |  | | | |  | | |  | | | | | |  |
|  | |  | | | |  | | | |  | | |  | | | | | |  |
|  | |  | | | |  | | | |  | | |  | | | | | |  |
|  | |  | | | |  | | | |  | | |  | | | | | |  |
|  | |  | | | |  | | | |  | | |  | | | | | |  |
| \|  \| \| --- \| | |  | | | |  | | | |  | | |  | | | | | |  |
|  | |  | | | |  | | | |  | | |  | | | | | |  |
|  | |  | | | |  | | | |  | | |  | | | | | |  |
|  | |  | | | |  | | | |  | | |  | | | | | |  |
|  | |  | | | |  | | | |  | | |  | | | | | |  |
|  | |  | | | |  | | | |  | | |  | | | | | |  |
|  | |  | | | |  | | | |  | | |  | | | | | |  |
|  | |  | | | |  | | | |  | | |  | | | | | |  |
|  | |  | | | |  | | | |  | | |  | | | | | |  |
|  | |  | | | |  | | | |  | | |  | | | | | |  |
|  | |  | | | |  | | | |  | | |  | | | | | |  |
|  | |  | | | |  | | | |  | | |  | | | | | |  |
|  | |  | | | |  | | | |  | | |  | | | | | |  |
|  | |  | | | |  | | | |  | | |  | | | | | |  |
|  | |  | | | |  | | | |  | | | 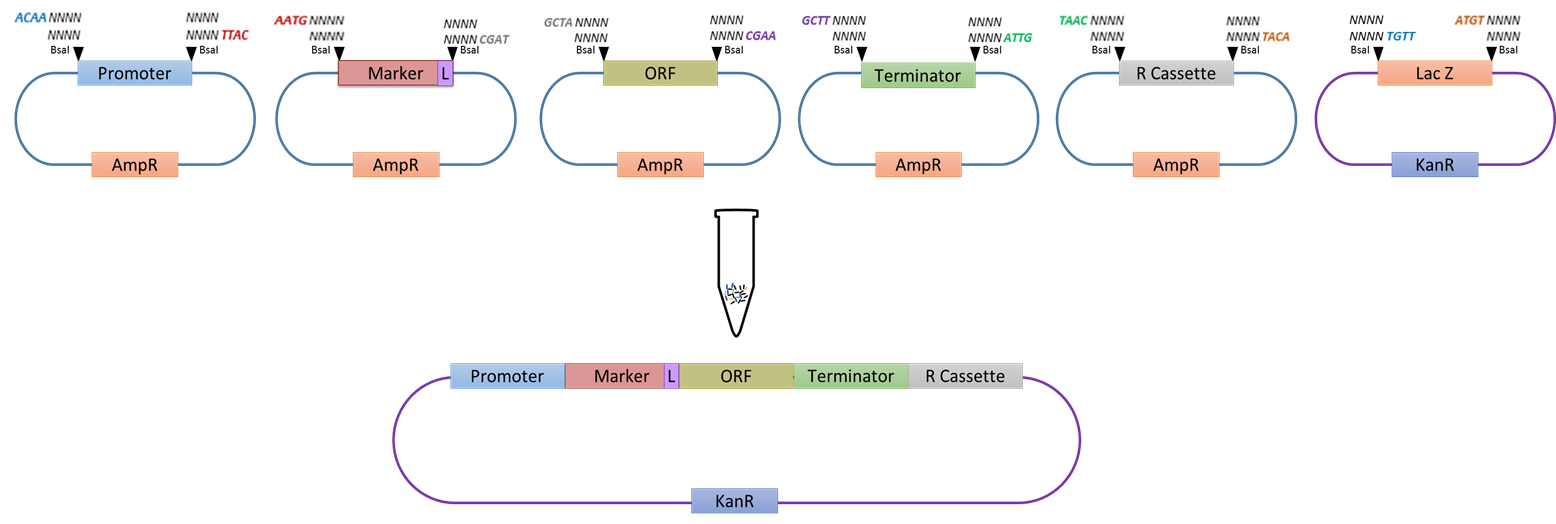 | | | | | |  |
| **Supplementary Table 3G**. TrichoGate strategy for C- terminal fusion tags | | | | | | | | | | | | | |  | |  | | | |
|  | | | |  | | | |  | | | | | | Recognition and cutting *Bsa*I site | | Target sequence | | | |
| **Promoter** | | | |  | | | |  | | | | | |  | |  | | | |
|  |  |  |  | Promoter X | | | | Left border-BsaI Forward | | | | | | GGTCTCt **ACAA** | | Sequence left region primer from promoter X | | | |
|  |  |  |  | Promoter X | | | | Left border-BsaI Reverse | | | | | | GGTCTCa **CATT** | | Reverse complementary left region primer from promoter X | | | |
|  |  |  |  |  | | | |  | | | | | |  | |  | | | |
| **Gene or marker** | | | |  | | | |  | | | | | |  | |  | | | |
|  |  |  |  | Marker or gene X | | | | Left region-BsaI Forward | | | | | | GGTCTCt **AATG** | | Sequence left region primer from gene X | | | |
|  |  |  |  | Marker or gene X | | | | Right region-BsaI Reverse | | | | | | GGTCTCc **CGGC** | | Reverse complementary left region primer from gene X | | | |
|  |  |  |  |  | | | |  | | | | | |  | |  | | | |
| **Tag X** | | | |  | | | |  | | | | | |  | |  | | | |
|  |  |  |  | Tag X | | | | Left region-BsaI Forward | | | | | | GGTCTCa **GCCG** | | Linker + sequence left region primer from tag X | | | |
|  |  |  |  | Tag X | | | | Right region-BsaI Reverse | | | | | | GGTCTCt **AAGC** | | Reverse complementary left region primer from tag X | | | |
|  |  |  |  |  | | | |  | | | | | |  | |  | | | |
| **Terminator** | | | |  | | | |  | | | | | |  | |  | | | |
|  |  |  |  | Terminator X | | | | Left region-BsaI Forward | | | | | | GGTCTCt **GCTT** | | Sequence left region primer from terminator X | | | |
|  |  |  |  | Terminator X | | | | Right region-BsaI Reverse | | | | | | GGTCTCc **GTTA** | | Reverse complementary left region primer from terminator X | | | |
|  |  |  |  |  | | | |  | | | | | |  | |  | | | |
| **Resistance cassette** | | | |  | | | |  | | | | | |  | |  | | | |
|  |  |  |  | Resistance cassette X | | | | Left region-BsaI Forward | | | | | | GGTCTCt **TAAC** | | Sequence left region primer from resistance cassette X | | | |
|  |  |  |  | Resistance cassette X | | | | Right region-BsaI Reverse | | | | | | GGTCTCa **ACAT** | | Reverse complementary left region primer from resistance cassette X | | | |
|  |  |  |  |  | | | |  | | | | | |  | |  | | | |
| **Example:** | | | | Primer name | | | |  | | | | | | Recognition and cutting *Bsa*I site | | Primer specific sequence (5'--->3') | | | |
|  | | | |  | | | |  | | | | | |  | |  | | | |
| Tef1 promoter | | | | Tef1 Fw | | | | Left region-BsaI Forward | | | | | | GGTCTCt **ACAA** | | TACCAACACAGAGGGACGCGC | | | |
| Tef1 promoter | | | | Tef1 Rw | | | | Right region-BsaI Reverse | | | | | | GGTCTCa **CATT** | | TTTTGCGGTTTGTGAAATG | | | |
| eGFP Marker | | | | eGFP Fw | | | | Left region-BsaI Forward | | | | | | GGTCTCt **AATG** | | CTCGAGATGGCTACTATG | | | |
| eGFP Marker | | | | eGFP (tags) Rw | | | | Right region-BsaI Reverse | | | | | | GGTCTCc **CGGC** | | TTACTTATACAACTCGTCCAT | | | |
| Tag HA | | | | 3xHA Fw | | | | Linker + left region-BsaI Reverse | | | | | | GGTCTCa **GCCG** | | CCTACCCCTATGACGTCCCTG | | | |
| Tag HA | | | | 3xHA Rw | | | | Right region-BsaI Reverse | | | | | | GGTCTCt **AAGC** | | CGGCGCGCCCTAGTAATCT | | | |
| T-nos terminator | | | | T-nos Fw | | | | Left region-BsaI Forward | | | | | | GGTCTCt **GCTT** | | GGCGCGCCGGCCGCCCGGC | | | |
| T-nos terminator | | | | T-nos Rw | | | | Right region-BsaI Reverse | | | | | | GGTCTCc **GTTA** | | AATTCTCATGTTTGACAGC | | | |
| HygromycinR | | | | HygR Fw | | | | Left region-BsaI Forward | | | | | | GGTCTCt **TAAC** | | CCGTTAACGGAACCCGGTCGG | | | |
| HygromycinR | | | | HygR Rw | | | | Right region-BsaI Reverse | | | | | | GGTCTCa **ACAT** | | TGATATTGAAGGAGCATTTTTG | | | |
|  | | | |  | | | |  | | | | | |  | |  | | | |
| Notes: red Colour lowercase nucleotide; any nucleotide of choice. N; represents any nucleotide depending on the sequence of the target DNA molecule. Colour nucleotides; represents four nucleotide overhangs. | | | | | | | | | | | | | | | | | | | |
| * For the reverse primers use the reverse complementary sequence of the desired gene | | | | | | | | | | | | | | | | | | | |
|  | | | |  | | | |  | | | | | |  | |  | | | |
|  | | | |  | | | |  | | | | | |  | |  | | | |
| \| 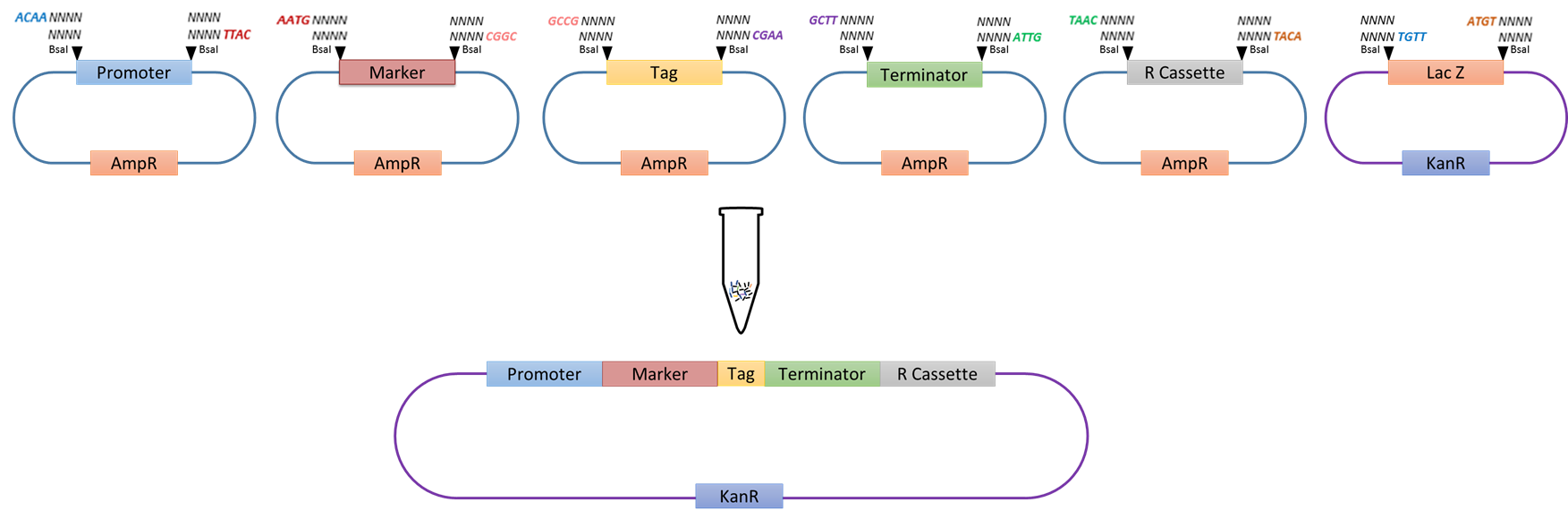 \| \| --- \| | | | |  | | | |  | | | | | |  | |  | | | |
|  | | | |  | | | |  | | | | | |  | |  | | | |
|  | | | |  | | | |  | | | | | |  | |  | | | |
|  | | | |  | | | |  | | | | | |  | |  | | | |
|  | | | |  | | | |  | | | | | |  | |  | | | |
|  | | | |  | | | |  | | | | | |  | |  | | | |
|  | | | |  | | | |  | | | | | |  | |  | | | |
|  | | | |  | | | |  | | | | | |  | |  | | | |
|  | | | |  | | | |  | | | | | |  | |  | | | |
|  | | | |  | | | |  | | | | | |  | |  | | | |
|  | | | |  | | | |  | | | | | |  | |  | | | |
|  | | | |  | | | |  | | | | | |  | |  | | | |
